# Impact of nationwide enhanced implementation of best practices in pancreatic cancer care (PACAP-1): a multicenter stepped-wedge cluster randomized controlled trial

**DOI:** 10.1186/s13063-020-4180-z

**Published:** 2020-04-16

**Authors:** T. M. Mackay, F. J. Smits, A. E. J. Latenstein, A. Bogte, B. A. Bonsing, H. Bos, K. Bosscha, L. A. A. Brosens, L. Hol, O. R. C. Busch, G. J. Creemers, W. L. Curvers, M. den Dulk, S. van Dieren, L. M. J. W. van Driel, S. Festen, E. J. M. van Geenen, L. G. van der Geest, D. J. A. de Groot, J. W. B. de Groot, N. Haj Mohammad, B. C. M. Haberkorn, J. T. Haver, E. van der Harst, G. J. M. Hemmink, I. H. de Hingh, C. Hoge, M. Y. V. Homs, N. C. van Huijgevoort, M. A. J. M. Jacobs, E. D. Kerver, M. S. L. Liem, M. Los, H. Lubbinge, S. A. C. Luelmo, V. E. de Meijer, L. Mekenkamp, I. Q. Molenaar, M. G. H. van Oijen, G. A. Patijn, R. Quispel, L. B. van Rijssen, T. E. H. Römkens, H. C. van Santvoort, J. M. J. Schreinemakers, H. Schut, T. Seerden, M. W. J. Stommel, A. J. ten Tije, N. G. Venneman, R. C. Verdonk, J. Verheij, F. G. I. van Vilsteren, J. de Vos-Geelen, A. Vulink, C. Wientjes, F. Wit, F. J. Wessels, B. Zonderhuis, C. H. van Werkhoven, J. E. van Hooft, C. H. J. van Eijck, J. W. Wilmink, H. W. M. van Laarhoven, M. G. Besselink, for the Dutch Pancreatic Cancer Group

**Affiliations:** 1 Department of surgery, Cancer Center Amsterdam, Amsterdam UMC, University of Amsterdam, PO Box 22660, 1100 DD Amsterdam, the Netherlands; 2 Department of surgery, University Medical Center Utrecht, Utrecht, the Netherlands; 3 Department of gastroenterology, Regional Academic Cancer Center Utrecht, University Medical Center Utrecht & St. Antonius Hospital, Nieuwegein, the Netherlands; 4 Department of surgery, Leiden University Medical Center, Leiden, the Netherlands; 5 Department of medical oncology, Tjongerschans Hospital, Heerenveen, the Netherlands; 6 Department of surgery, Jeroen Bosch Hospital, Den Bosch, the Netherlands; 7 Department of pathology, University Medical Center Utrecht, Utrecht, the Netherlands; 8 Department of pathology, Radboud University, Nijmegen, the Netherlands; 9 Department of gastroenterology, Maasstad Hospital, Rotterdam, the Netherlands; 10 Department of medical oncology, Catharina Hospital, Eindhoven, the Netherlands; 11 Department of gastroenterology, Catharina Hospital, Eindhoven, the Netherlands; 12 Department of surgery, Maastricht UMC+, Maastricht, the Netherlands; 13 Department of gastroenterology, Erasmus Medical Center, Rotterdam, the Netherlands; 14 Department of surgery, OLVG, Amsterdam, the Netherlands; 15 Department of gastroenterology, Radboud UMC, Nijmegen, the Netherlands; 16 Department of Research, Netherlands Comprehensive Cancer Organisation (IKNL), Utrecht, the Netherlands; 17 Department of medical oncology, University Medical Center Groningen, Groningen, the Netherlands; 18 Department of medical oncology, Oncology Center Isala, Zwolle, the Netherlands; 19 Department of Medical Oncology, Regional Academic Cancer Center Utrecht, University Medical Center Utrecht & St. Antonius Hospital, Nieuwegein, the Netherlands; 20 Department of medical oncology, Maasstad Hospital, Rotterdam, the Netherlands; 21 Department of nutrition and dietetics, Cancer Center Amsterdam, Amsterdam UMC, University of Amsterdam, Amsterdam, the Netherlands; 22 Department of surgery, Maasstad Hospital, Rotterdam, the Netherlands; 23 Department of gastroenterology, Oncology Center Isala, Zwolle, the Netherlands; 24 Department of surgery, Catharina Hospital, Eindhoven, the Netherlands; 25 Department of gastroenterology, Maastricht UMC+, Maastricht, the Netherlands; 26 Department of medical oncology, Erasmus Medical Center, Rotterdam, the Netherlands; 27 Department of gastroenterology, Cancer Center Amsterdam, Amsterdam UMC, University of Amsterdam, Amsterdam, the Netherlands; 28 Department of gastroenterology, Cancer Center Amsterdam, Amsterdam UMC, VU Medical Center, Amsterdam, the Netherlands; 29 Department of medical oncology, OLVG, Amsterdam, the Netherlands; 30 Department of surgery, Medisch Spectrum Twente, Enschede, the Netherlands; 31 Department of gastroenterology, Tjongerschans Hospital, Heerenveen, the Netherlands; 32 Department of medical oncology, Leiden University Medical Center, Leiden, the Netherlands; 33 Department of surgery, University Medical Center Groningen, Groningen, the Netherlands; 34 Department of medical oncology, Medisch Spectrum Twente, Enschede, the Netherlands; 35 Department of surgery, Regional Academic Cancer Center Utrecht, University Medical Center Utrecht & St. Antonius Hospital, Nieuwegein, the Netherlands; 36 Department of medical oncology, Cancer Center Amsterdam, Amsterdam UMC, University of Amsterdam, Amsterdam, the Netherlands; 37 Department of surgery, Oncology Center Isala, Zwolle, the Netherlands; 38 Department of gastroenterology, Reinier de Graaf Hospital, Delft, the Netherlands; 39 Department of gastroenterology, Jeroen Bosch Hospital, Den Bosch, the Netherlands; 40 Department of surgery, Amphia Hospital, Breda, the Netherlands; 41 Department of medical oncology, Jeroen Bosch Hospital, Den Bosch, the Netherlands; 42 Department of gastroenterology, Amphia Hospital, Breda, the Netherlands; 43 Department of surgery, Radboud UMC, Nijmegen, the Netherlands; 44 Department of medical oncology, Amphia Hospital, Breda, the Netherlands; 45 Department of gastroenterology and hepatology, Medisch Spectrum Twente, Enschede, the Netherlands; 46 Department of pathology, Cancer Center Amsterdam, Amsterdam UMC, University of Amsterdam, Amsterdam, the Netherlands; 47 Department of gastroenterology, University Medical Center Groningen, Groningen, the Netherlands; 48 Department of medical oncology, Maastricht UMC+, Maastricht, the Netherlands; 49 Department of medical oncology, Reinier de Graaf Hospital, Delft, the Netherlands; 50 Department of gastroenterology, OLVG, Amsterdam, the Netherlands; 51 Department of surgery, Tjongerschans Hospital, Heerenveen, the Netherlands; 52 Department of radiology, Regional Academic Cancer Center Utrecht, University Medical Center Utrecht & St. Antonius Hospital, Nieuwegein, the Netherlands; 53 Department of surgery, Cancer Center Amsterdam, Amsterdam UMC, VU Medical Center, Amsterdam, the Netherlands; 54 Julius Center for Health Sciences and primary care, University Medical Center Utrecht, Utrecht University, Utrecht, the Netherlands; 55 Department of surgery, Erasmus Medical Center, Rotterdam, the Netherlands

**Keywords:** Pancreatic cancer, Survival, Quality of life, Stepped-wedge cluster randomized controlled trial, Implementation, Best practices, Chemotherapy, Biliary drainage, Pancreatic enzyme replacement therapy, Registry

## Abstract

**Background:**

Pancreatic cancer has a very poor prognosis. Best practices for the use of chemotherapy, enzyme replacement therapy, and biliary drainage have been identified but their implementation in daily clinical practice is often suboptimal. We hypothesized that a nationwide program to enhance implementation of these best practices in pancreatic cancer care would improve survival and quality of life.

**Methods/design:**

PACAP-1 is a nationwide multicenter stepped-wedge cluster randomized controlled superiority trial. In a per-center stepwise and randomized manner, best practices in pancreatic cancer care regarding the use of (neo)adjuvant and palliative chemotherapy, pancreatic enzyme replacement therapy, and metal biliary stents are implemented in all 17 Dutch pancreatic centers and their regional referral networks during a 6-week initiation period. Per pancreatic center, one multidisciplinary team functions as reference for the other centers in the network. Key best practices were identified from the literature, 3 years of data from existing nationwide registries within the Dutch Pancreatic Cancer Project (PACAP), and national expert meetings. The best practices follow the Dutch guideline on pancreatic cancer and the current state of the literature, and can be executed within daily clinical practice. The implementation process includes monitoring, return visits, and provider feedback in combination with education and reminders. Patient outcomes and compliance are monitored within the PACAP registries. Primary outcome is 1-year overall survival (for all disease stages). Secondary outcomes include quality of life, 3- and 5-year overall survival, and guideline compliance. An improvement of 10% in 1-year overall survival is considered clinically relevant. A 25-month study duration was chosen, which provides 80% statistical power for a mortality reduction of 10.0% in the 17 pancreatic cancer centers, with a required sample size of 2142 patients, corresponding to a 6.6% mortality reduction and 4769 patients nationwide.

**Discussion:**

The PACAP-1 trial is designed to evaluate whether a nationwide program for enhanced implementation of best practices in pancreatic cancer care can improve 1-year overall survival and quality of life.

**Trial registration:**

ClinicalTrials.gov, NCT03513705. Trial opened for accrual on 22th May 2018.

## Administrative information

Note: the numbers in curly brackets in this protocol refer to SPIRIT checklist item numbers. The order of the items has been modified to group similar items (see http://www.equator-network.org/reporting-guidelines/spirit-2013-statement-defining-standard-protocol-items-for-clinical-trials/).

**Table:** 

**Title {1}**	Impact of nationwide enhanced implementation of best practices in pancreatic cancer care (PACAP-1): a multicenter stepped-wedge cluster randomized controlled trial
**Trial registration {2a and 2b}.**	Trial open for accrual 22th May 2018. ClinicalTrials.gov - NCT03513705.
**Protocol version {3}**	Protocol version 6.4 – May 2018
**Funding {4}**	This research was funded by a grant from the Dutch Cancer Society (grant number UVA2013-5842).
**Author details {5a}**	**TM Mackay:** Department of surgery, Cancer Center Amsterdam, Amsterdam UMC, University of Amsterdam **FJ Smits:** Department of surgery, University Medical Center Utrecht, Utrecht **AEJ Latenstein:** Department of surgery, Cancer Center Amsterdam, Amsterdam UMC, University of Amsterdam **A Bogte:** Department of gastroenterology, Regional Academic Cancer Center Utrecht, University Medical Center Utrecht & St. Antonius Hospital Nieuwegein **BA Bonsing:** Department of surgery, Leiden University Medical Center, Leiden **H Bos:** Department of medical oncology, Tjongerschans Hospital, Heerenveen **K Bosscha:** Department of surgery, Jeroen Bosch Hospital, Den Bosch **LAA Brosens:** Department of pathology, University Medical Center Utrecht, Utrecht; and Department of pathology, Radboud University, Nijmegen **L Hol:** Department of gastroenterology, Maasstad Hospital, Rotterdam **ORC Busch:** Department of surgery, Cancer Center Amsterdam, Amsterdam UMC, University of Amsterdam **GJ Creemers:** Department of medical oncology, Catharina Hospital, Eindhoven **WL Curvers:** Department of gastroenterology, Catharina Hospital, Eindhoven **M den Dulk:** Department of surgery, Maastricht UMC+, Maastricht **S van Dieren:** Department of surgery, Cancer Center Amsterdam, Amsterdam UMC, University of Amsterdam **LMJW van Driel:** Department of gastroenterology, Erasmus Medical Center, Rotterdam **S Festen:** Department of surgery, OLVG, Amsterdam **EJM van Geenen:** Department of gastroenterology, Radboud UMC, Nijmegen **LG van der Geest:** Department of Research, Netherlands Comprehensive Cancer Organisation (IKNL), Utrecht **DJA de Groot:** Department of medical oncology, University Medical Center Groningen **JWB de Groot:** Department of medical oncology, Oncology Center Isala, Zwolle **N Haj Mohammad:** Department of medical oncology, Regional Academic Cancer Center Utrecht, University Medical Center Utrecht & St. Antonius Hospital Nieuwegein **BCM Haberkorn:** Department of medical oncology, Maasstad Hospital, Rotterdam **JT Haver:** Department of nutrition and dietetics, Cancer Center Amsterdam, Amsterdam UMC, University of Amsterdam **E van der Harst:** Department of surgery, Maasstad Hospital, Rotterdam **GJM Hemmink:** Department of gastroenterology, Oncology Center Isala, Zwolle **IH de Hingh:** Department of surgery, Catharina Hospital, Eindhoven **C Hoge:** Department of gastroenterology, Maastricht UMC+, Maastricht **MYV Homs:** Department of medical oncology, Erasmus Medical Center, Rotterdam **NC van Huijgevoort:** Department of gastroenterology, Cancer Center Amsterdam, Amsterdam UMC, University of Amsterdam **MAJM Jacobs:** Department of gastroenterology, Cancer Center Amsterdam, Amsterdam UMC, VU Medical Center **ED Kerver:** Department of medical oncology, OLVG, Amsterdam **MSL Liem:** Department of surgery, Medisch Spectrum Twente, Enschede **M Los:** Department of medical oncology, Regional Academic Cancer Center Utrecht, University Medical Center Utrecht & St. Antonius Hospital Nieuwegein **H Lubbinge:** Department of gastroenterology, Tjongerschans Hospital, Heerenveen **SAC Luelmo:** Department of medical oncology, Leiden University Medical Center, Leiden **VE de Meijer:** Department of surgery, University Medical Center Groningen, Groningen **L Mekenkamp:** Department of medical oncology, Medisch Spectrum Twente, Enschede **IQ Molenaar:** Department of surgery, Regional Academic Cancer Center Utrecht, University Medical Center Utrecht & St. Antonius Hospital Nieuwegein **MGH van Oijen:** Department of medical oncology, Cancer Center Amsterdam, Amsterdam UMC, University of Amsterdam **GA Patijn:** Department of surgery, Oncology Center Isala, Zwolle **R Quispel:** Department of gastroenterology, Reinier de Graaf Hospital, Delft **LB van Rijssen:** Department of surgery, Cancer Center Amsterdam, Amsterdam UMC, University of Amsterdam **TEH Römkens:** Department of gastroenterology, Jeroen Bosch Hospital, Den Bosch **HC van Santvoort:** Department of surgery, Regional Academic Cancer Center Utrecht, University Medical Center Utrecht & St. Antonius Hospital Nieuwegein **JMJ Schreinemakers:** Department of surgery, Amphia Hospital, Breda **H Schut:** Department of medical oncology, Jeroen Bosch Hospital, Den Bosch **T Seerden:** Department of gastroenterology, Amphia Hospital, Breda **MWJ Stommel:** Department of surgery, Radboud UMC, Nijmegen **AJ ten Tije:** Department of medical oncology, Amphia Hospital, Breda **NG Venneman:** Department of gastroenterology and hepatology, Medisch Spectrum Twente, Enschede **RC Verdonk:** Department of gastroenterology, Regional Academic Cancer Center Utrecht, University Medical Center Utrecht & St. Antonius Hospital Nieuwegein **J Verheij:** Department of pathology, Cancer Center Amsterdam, Amsterdam UMC, University of Amsterdam **FGI van Vilsteren:** Department of gastroenterology, University Medical Center Groningen, Groningen **J de Vos-Geelen:** Department of medical oncology, Maastricht UMC+, Maastricht **A Vulink:** Department of medical oncology, Reinier de Graaf Hospital, Delft **C Wientjes:** Department of gastroenterology, OLVG, Amsterdam **F Wit:** Department of surgery, Tjongerschans Hospital, Heerenveen **FJ Wessels:** Department of radiology, Regional Academic Cancer Center Utrecht, University Medical Center Utrecht & St. Antonius Hospital Nieuwegein **B Zonderhuis:** Department of surgery, Cancer Center Amsterdam, Amsterdam UMC, VU Medical Center **CH van Werkhoven:** Julius Center for Health Sciences and primary care, University Medical Center Utrecht, Utrecht University **JE van Hooft:** Department of gastroenterology, Cancer Center Amsterdam, Amsterdam UMC, University of Amsterdam **CHJ van Eijck:** Department of surgery, Erasmus Medical Center, Rotterdam **JW Wilmink:** Department of medical oncology, Cancer Center Amsterdam, Amsterdam UMC, University of Amsterdam **HWM van Laarhoven:** Department of medical oncology, Cancer Center Amsterdam, Amsterdam UMC, University of Amsterdam **MG Besselink:** Department of surgery, Cancer Center Amsterdam, Amsterdam UMC, University of Amsterdam
**Name and contact information for the trial sponsor {5b}**	Dutch Cancer Society (https://www.kwf.nl/node/20)
**Role of sponsor {5c}**	The Dutch Cancer Society played no role in the design of the study and collection, analysis, and interpretation of data and in writing the manuscript.

## Background {a}

It is estimated that pancreatic cancer will be the second most common cause of cancer-related mortality by 2030 in Europe [1]. Without treatment, the median survival is only 3 to 6 months. Some 15–20% of patients with pancreatic cancer are amenable to surgical resection combined with adjuvant chemotherapy [2]. However, even after resection, the median overall survival is only 11–25 months [1, 3]. In patients in whom it is possible to perform a microscopic radical resection median survival increases to 3 to 4 years [3–5].

### The Dutch Pancreatic Cancer Project

The Dutch Pancreatic Cancer Project (PACAP) aims to improve outcomes of patients in all stages of pancreatic cancer. PACAP was launched in 2013 as an initiative of the national multidisciplinary Dutch Pancreatic Cancer Group (DPCG, www.dpcg.nl) [6]. In a period of 6 years, PACAP aimed primarily to improve outcome and quality of life for pancreatic cancer patients in the Netherlands. This is achieved through one of the largest nationwide collaborative outcomes registration and biobanking projects on pancreatic cancer in the world, which provides unique opportunities for improving care for these patients and developing new diagnostic and treatment strategies. From the start, PACAP included several registries, including the Dutch Pancreatic Cancer Audit (DPCA), the Netherlands Cancer Registry (NCR), the Dutch Pancreas Biobank (PancreasParel), patient-reported outcome measures (PROMs), and an online expert panel [7–9]. Details on PACAP registries are listed in Appendix 1.

### The PACAP-1 trial {6b} {15}

**Fig. 1:**
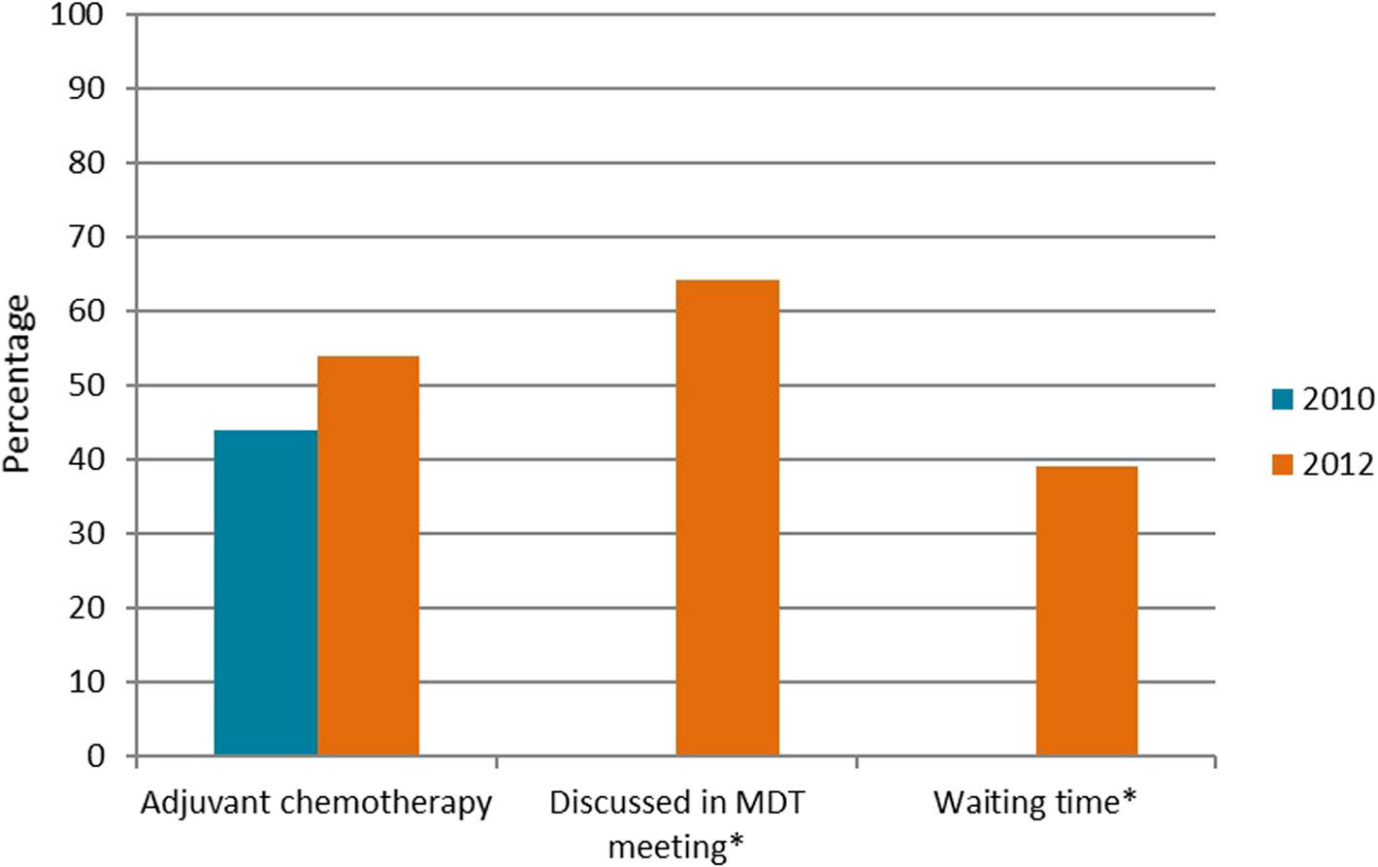
Guideline compliance among 2564 patients treated for pancreatic or periampullary cancer in the Netherlands in 2010 and 2012. *MDT* multidisciplinary team. *Adjuvant chemotherapy*, percentage of patients receiving adjuvant chemotherapy after tumor resection for pancreatic carcinoma. *Discussed in MDT meeting*, percentage of patients discussed within a MDT meeting. *Waiting time*, percentage of patients who started curative treatment within three weeks of final MDT meeting. * Not available for 2010

**Fig. 2:**
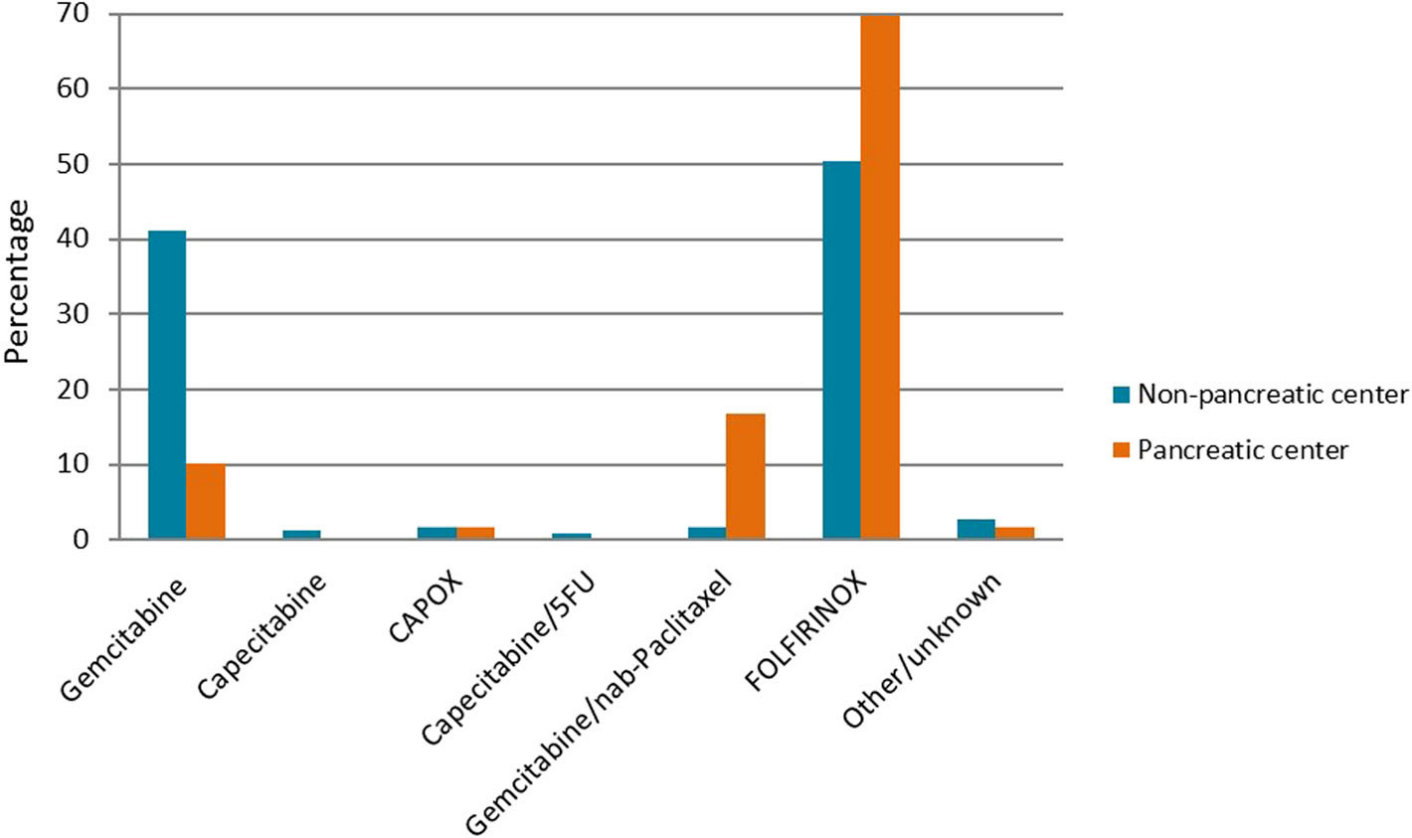
Type of palliative chemotherapy given to 345 patients with metastasized pancreatic cancer in 2015 in the Netherlands in pancreatic and non-pancreatic centers (NCR data). *CAPOX* capecitabine and oxaliplatin, *5FU* 5-fluorouracil, *FOLFIRINOX* folinic acid, 5-fluorouracil, irinotecan, and oxaliplatin

In 2014, 78% of 2393 patients diagnosed with pancreatic cancer in the Netherlands died within 1 year (www.cijfersoverkanker.nl). These numbers illustrate the severity of this disease and the need for improvement of treatment and clinical outcomes. From the literature and the first 3 years of PACAP, fairly straightforward points of improvement in care and guideline compliance for patients with pancreatic cancer in the Netherlands were identified. Systematic reviews of guideline dissemination and implementation strategies showed that compliance by health-care workers, specifically medical doctors, is poor [10, 11]. A recent study demonstrated that compliance with the 2012 Dutch pancreatic cancer guideline was low (Fig. 1) [12]. In addition, regional differences in (type of) treatment and clinical outcomes have been identified. For example, the use of adjuvant chemotherapy after pancreatoduodenectomy for pancreatic cancer per DPCG center varied between 26 and 74% in 1195 Dutch patients (2008–2013) [13]. Significant differences were also present in the type of palliative chemotherapy given to 345 patients with metastatic disease (Fig. 2). Patients with metastatic disease who were treated in high-volume chemotherapy or surgical centers had better survival compared to lower volume centers [14]. While administration of palliative systemic chemotherapy doubled in the elderly in the Netherlands between 2005 and 2013 (13% vs. 30%), it was still relatively low compared with population-based studies from other western countries [15].

The PACAP-1 trial aims to enhance the implementation of key best practices in the 17 Dutch pancreatic centers with their associated regional networks, using a nationwide stepped-wedge cluster randomized controlled trial (RCT). PACAP-1 is unique in that it involves all relevant medical specialties and all Dutch hospitals treating patients with pancreatic cancer. PACAP-1 will use the registries already included in PACAP to audit current practice and improve adherence to best practices and synoptic reporting in the Netherlands for pancreatic cancer patients, including the Dutch evidence-based guideline on pancreatic cancer [16]. Most importantly, with the PACAP infrastructure, the levels of implementation and compliance and the effect on patient outcomes can be assessed. We hypothesize that survival and quality of life will improve for pancreatic cancer patients in the Netherlands by a program to enhance implementation of best practices.

## Methods/design

### Study setting {9}

The PACAP-1 trial will implement best practices in and collect data from all hospitals (e.g., academic, top-clinical, general) in the Netherlands. A list of the DPCG centers where pancreatic surgery is performed can be found at www.dpcg.nl.

### Primary aim {7}

The primary aim of PACAP-1 is to evaluate whether a nationwide program for enhanced implementation of best practices can improve 1-year overall survival by 10% in all pancreatic cancer patients in the Netherlands. Ten percent was considered to be clinically relevant.

### Secondary aims {7}

Secondary aims are to evaluate whether enhanced implementation of key best practices can improve quality of life (main secondary objective) and clinical outcomes (3- and 5-year overall survival and treatment complications). Another aim is to improve the use of nationwide standardized “best practice” reports by radiologists, surgeons, pathologists, medical oncologists, and gastroenterologists. Hereby, we aim to optimize data registry with key parameter and synoptic reporting that will lead to efficient and high-quality data collection. Finally, we aim to improve participation in DPCG RCTs, especially those which aim to improve survival and/or quality of life.

### PACAP-1 trial design {8} {13}

**Fig. 3:**
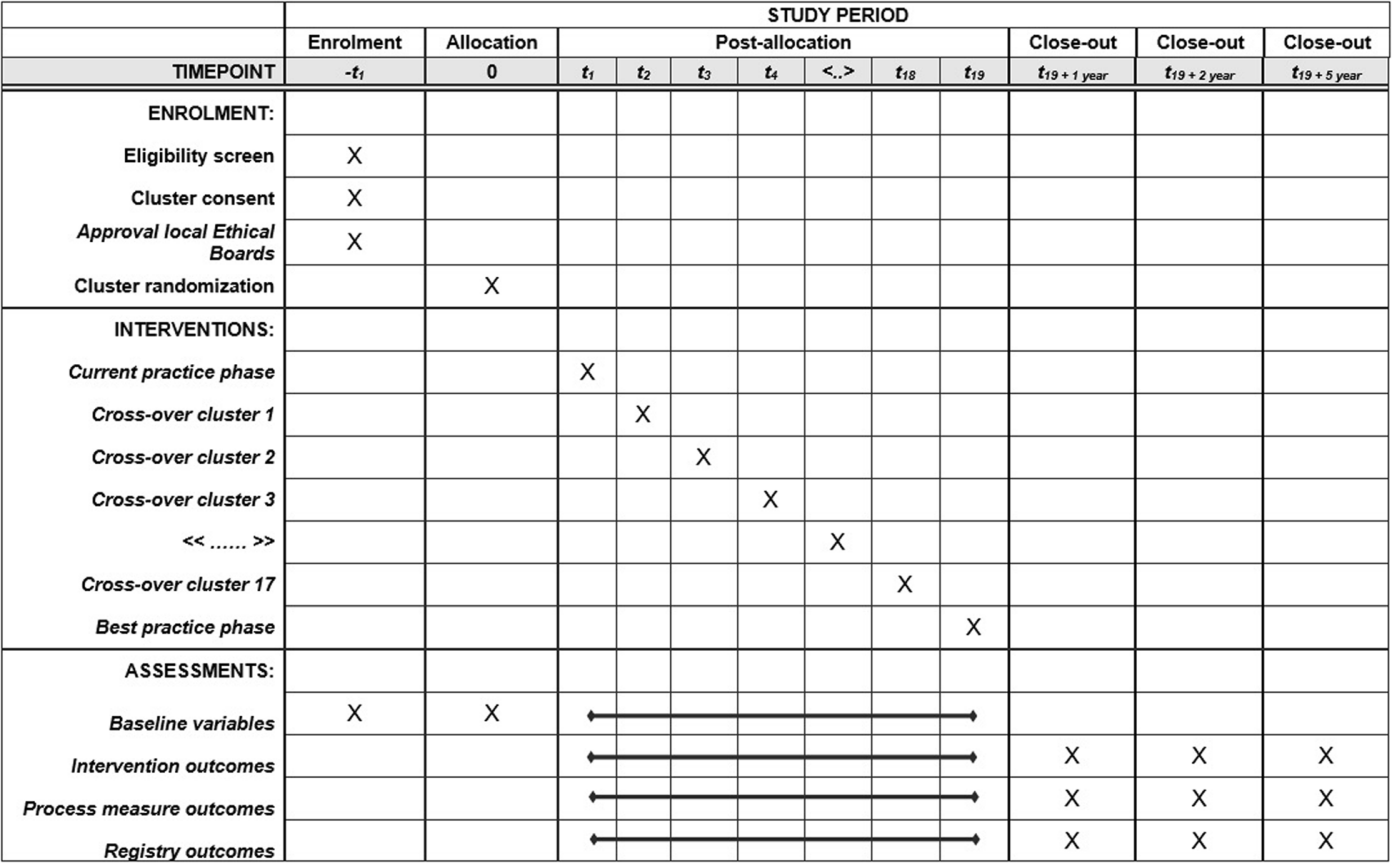
Schedule of enrolment, interventions, and assessments of PACAP-1 according to SPIRIT guidelines

The PACAP-1 trial is a nationwide stepped-wedge cluster RCT which aims for enhanced implementation of best practices in all 17 DPCG pancreatic cancer centers and their respective referral networks. Per pancreatic center and network, one regional pancreatic cancer team serves as reference for the other centers in the network. The pancreatic cancer team included at least a medical oncologist, a gastroenterologist, and a surgeon, regularly together with a specialized nurse. This trial was designed in adherence to the CONSORT statement for cluster randomized trials [17] and extension for stepped-wedge trials [18], and SPIRIT guidelines for clinical trials [19]. For an overview of PACAP-1, see the SPIRIT figure (Fig. 3) and the SPIRIT checklist (Supplementary materials).

**Fig. 4:**
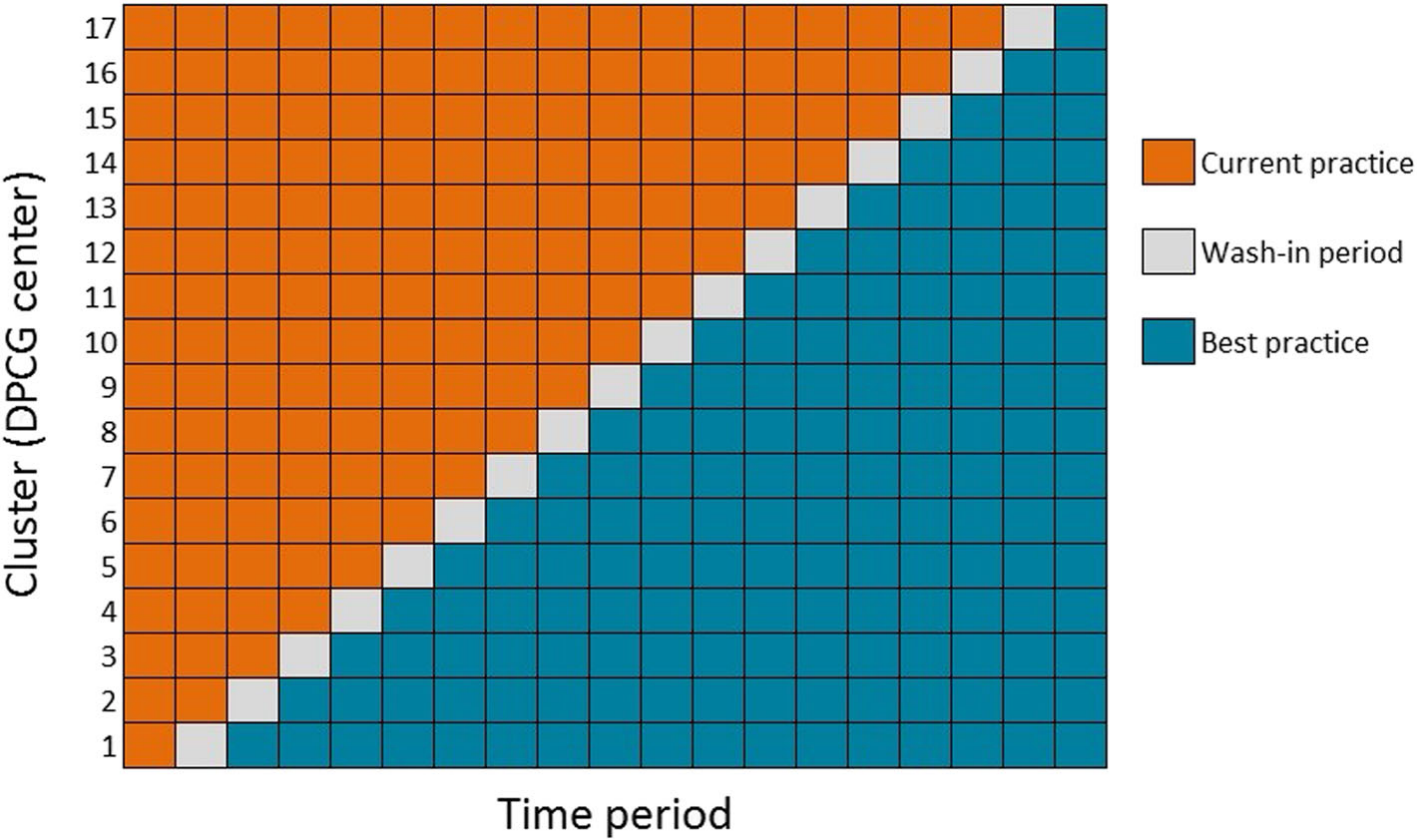
Schematic representation of PACAP-1 stepped-wedge cluster randomized controlled trial. *DPCG* Dutch Pancreatic Cancer Group

**Fig. 5:**
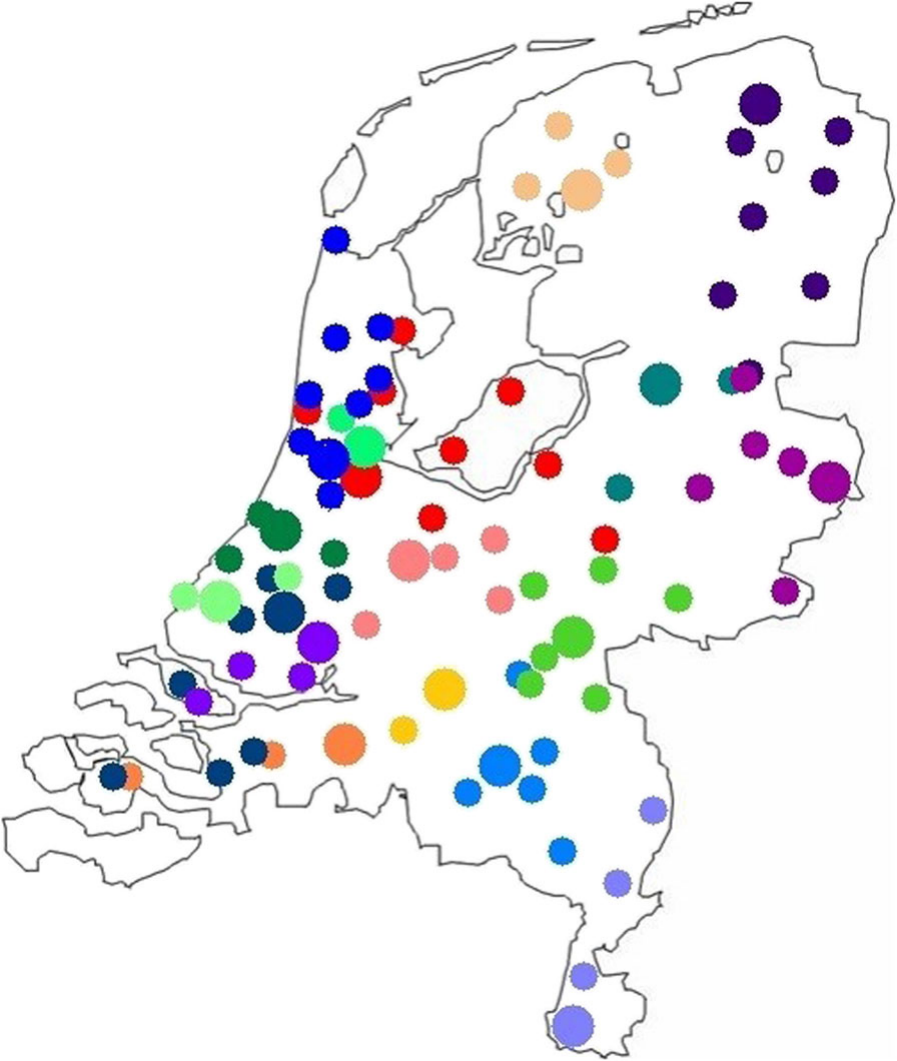
Schematic representation of 17 Dutch Pancreatic Cancer Group centers (*large dots*) and their respective referral networks and centers (*smaller dots*) per color. Note that referral centers may refer patients to more than one pancreatic center and therefore this figure is only for illustration

A schematic overview of the stepped-wedge trial design is provided in Fig. 4. In a step-wise manner, each cluster will cross-over from control (current practice) to intervention (best practice) phase. Each cluster contains one DPCG center and its referral region (Fig. 5), and therefore the number of sequences is equal to the number of participating centers. At the start of the study all clusters will be in the control phase. After 25 months, all 17 clusters will have crossed over to the intervention phase.

The duration of the trial is determined by the required sample size. Details of the sample size calculation are described in the “Sample size calculation” section. The order in which the clusters will cross-over is randomized [20, 21].

To achieve effective implementation of PACAP-1 best practices, a structured 6-week wash-in phase was designed (Appendix 2). Also, in this timeframe the study team will discuss with the local pancreatic cancer team how to implement best practices efficiently. It is important to avoid contamination of best practice for clusters still in the control phase. Therefore, details on PACAP-1 best practices will not be shared with local clinicians before the transfer to the intervention phase. In the analysis of PACAP-1, every cluster is their own control because of the cluster RCT design.

### Study population {10} {15}

All patients with pancreatic cancer in the Netherlands.

### Patient inclusion criteria {10}

Patients with pathologically or clinically diagnosed pancreatic ductal adenocarcinoma, all ages and all stages.

### Patient exclusion criteria {10}

None.

### Center inclusion criteria {10}

All 17 centers of the DPCG with their respective referral network. Each DPCG center performs at least 20 pancreatoduodenectomies (PDs) annually. Each center already has a coordinating role for pancreatic cancer for its regional network (Fig. 5). It is expected that the enhanced implementation of best practices will have an impact in the entire local network. A survey was conducted among DPCG centers to identify peripheral centers that mainly refer to their DPCG center. Outcomes of this survey were checked with NCR data and discrepancies only occurred for two centers. With these centers and the particular DPCG centers, it was discussed in what region the center would fit best.

### Center exclusion criteria {10}

There are no specific center exclusion criteria.

### Study endpoints {12}

#### Primary endpoint

The primary endpoint is 1-year overall survival.

#### Secondary endpoints

Secondary study endpoints are divided into intervention (e.g., quality of life, 3- and 5- year survival, and treatment complications such as chemotherapy toxicity), process measure (e.g., proportion of post-pancreatectomy patients receiving adjuvant chemotherapy, and proportion of patients requiring biliary drainage receiving a metal stent), registry (e.g., proportion of patients registered for PROMs or in DPCA, and proportion of patients where the CT scan checklist was used), and other outcomes (e.g., proportion of patients included in other DPCG prospective trials). See Supplementary materials for a detailed list of the secondary endpoints.

### Sample size calculation {14}

PACAP-1 is a superiority trial with 1-year overall survival as primary endpoint, which will be extracted from NCR survival data. The sample size calculation was based on the data from Table 1.

**Table 1:** Unpublished data from the Netherlands Cancer Registry of new patients diagnosed with pancreatic cancer in the year 2014

New patients diagnosed in DPCG centers	1075
One-year mortality rate in DPCG centers	702/1075 65%
New patients in the Netherlands	2393
One-year mortality rate in the Netherlands	1855/2393 78%
Intra-cluster coefficient (95% CI) between DPCG centers for one-year mortality	Approach A^1^: 0.0185 (0.0132–0.0575) Approach B^2^: 0.0183 (0.0131–0.0560)

^1^ Method A from the AOD library in R uses generalized linear mixed model

^2^ Method B from the AOD library in R uses generalized linear mixed model with Monte Carlo simulations

The required sample size was calculated using the formula for stepped-wedge designs [22]. Sample sizes were calculated for different effect sizes, different intra-cluster coefficients, for 80% or 90% power, and for the DPCG centers and for all of the Netherlands separately, using a cluster autocorrelation (CAC) of 1 [23] and a two-sided alpha of 0.05 (Table 2). Subsequently, it was reverse calculated which effect sizes could be determined with 80% and 90% power given a fixed study duration (hence a fixed sample size) of 25 months for the different other assumptions (Table 2). For logistical reasons inherent to successful implementation of different (discipline transcending) interventions, a shorter study duration was not considered.

**Table 2:** Power for effect size given fixed sample size

Population	N	p0	p1	RD	ICC	Power	Interpretation
*25-month study duration (including 5.8-week wash-in period)*							
DPCG	2142	0.65	0.550	− 0.100	0.0184	0.8	80% power for true reduction of 10.0%
DPCG	2142	0.65	0.535	− 0.115	0.0184	0.9	90% power for true reduction of 11.5%
All NL	4769	0.78	0.714	− 0.066	0.0368	0.8	80% power for true reduction of 6.6%
All NL	4769	0.78	0.704	− 0.076	0.0368	0.9	90% power for true reduction of 7.6%
All NL	4769	0.78	0.722	− 0.058	0.0092	0.8	80% power for true reduction of 5.8%
All NL	4769	0.78	0.712	− 0.068	0.0092	0.9	90% power for true reduction of 6.8%

*N* sample size, *p0* current 1-year mortality, *p1* expected 1-year mortality, *RD* risk difference, *ICC* intra-cluster correlation coefficient, *CAC* cluster autocorrelation, *DPCG* Dutch Pancreatic Cancer Group, *NL* the Netherlands

An improvement of 10% in 1-year overall survival for all patients with pancreatic cancer in the Netherlands is considered clinically relevant, and could be established following the PACAP-1 interventions. A 25-month study duration was chosen, which provides 80% statistical power for an absolute mortality reduction of 10.0% and 90% power for a reduction of 11.5% in the 17 pancreatic cancer centers, with a required sample size of 2142 patients. For all of the Netherlands, assuming the intracluster correlation coefficient (ICC) will be higher, the corresponding sample size provides 80% power for an absolute mortality reduction of 6.6% and 90% power for a reduction of 7.6% (Table 2).

### Intervention phase: PACAP-1 best practices {11a}

**Fig. 6:**
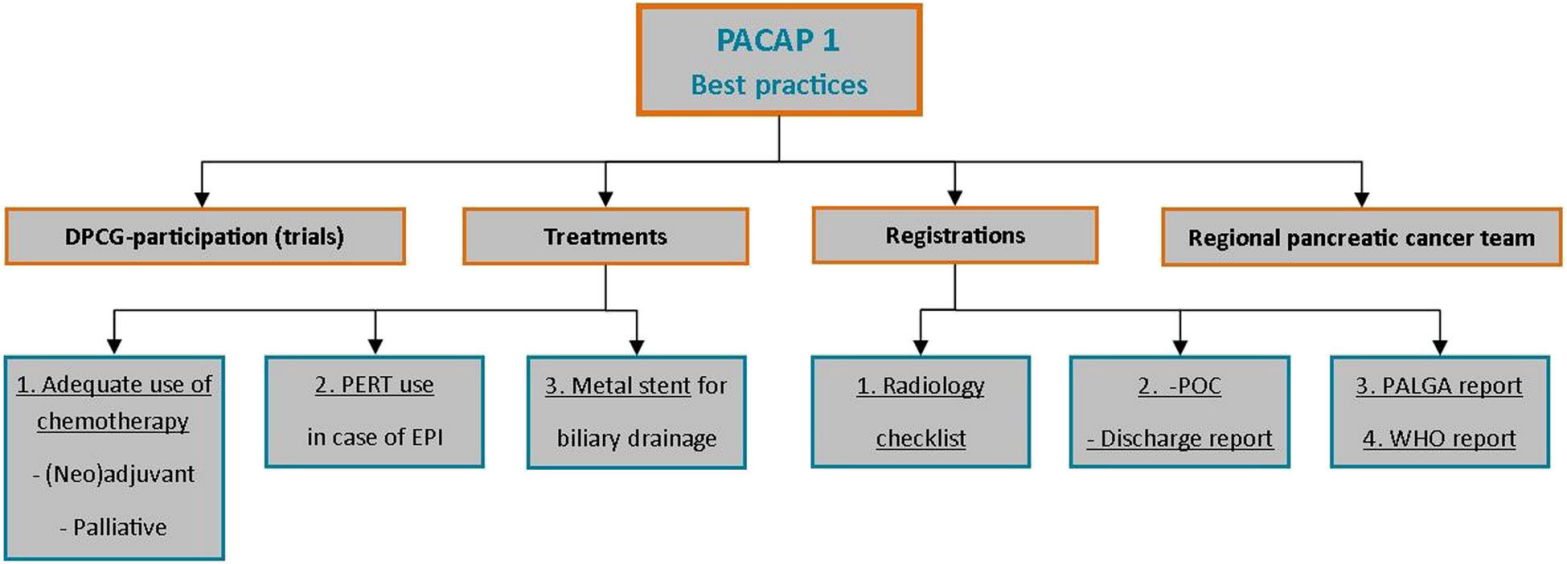
Schematic representation of PACAP-1 best practices. *PERT* pancreatic enzyme replacement therapy, *EPI* exocrine pancreatic insufficiency, *POC* postoperative conclusion, *PALGA* nationwide network and registry of histo- and cytopathology of the Netherlands, *WHO* World Health Organization performance status

To determine key best practices, points of improvement for three key medical specialties (medical oncology, gastroenterology, and surgery) were identified from literature and the first 3 years of PACAP (July 2014–July 2017). These are divided into intervention and registry categories (Fig. 6). Best-practice treatments aim to improve survival, clinical outcomes, and quality of life. Best-practice registrations aim to optimize data registry with key parameter and synoptic reporting that will lead to efficient and high-quality data collection. PACAP-1 interventions are listed in Appendix 3 in Table 3 per medical specialism. An overview of PACAP projects is presented in Appendix 1. Background and details per best practice are found in the Supplementary materials.

#### Best practice treatments

All treatments follow the current the Dutch guideline on pancreatic cancer [16] and the literature.

Treatment-1: Optimal patient information and use of (neoadjuvant, adjuvant, and palliative) chemotherapy.

Treatment-2: Pancreatic enzyme replacement therapy (PERT) and referral to dietician in case of exocrine pancreatic insufficiency (EPI).

Treatment-3: Metal stents for biliary drainage.

#### Best practice registration

Registration-1: Use of checklist for radiology reports of pancreatic cancer.

Registration-2: Use of standardized table with intra-operative events in operation report and complications of surgical treatment in discharge letters.

Registration-3: Use of nationwide standard for synoptic reporting of pancreatic cancer pathology from PALGA, the nationwide network and registry of histo- and cytopathology of the Netherlands.

Registration-4: Report of World Health Organization (WHO) performance status.

#### Additional best practices

Other-1: Inclusion of pancreatic cancer patients in PACAP PROMs registry.

Other-2: Participation in PancreasParel biobank.

Other-3: Pathologic confirmation in patients with (suspected) metastatic and locally advanced pancreatic cancer (LAPC).

Other-4: Participation in DPCG RCTs.

### Control phase: current practices

Current practice will be left to the discretion of the healthcare providers in the control phase. Centers will not learn the details of the best practices until the 6-week wash-in period of their region.

### National expert meeting

In preparation of the PACAP-1 trial, a national expert meeting was organized for one oncologist and/or one surgeon per DPCG center to improve support and buy-in, and to optimize the design of the trial including the three intervention best practices (i.e., 1) optimizing chemotherapy, 2) EPI treatment, and 3) biliary drainage with metal stents). To minimize contamination in the study we chose to invite only one specialist per center. Oncologists and surgeons working in 11 DPCG centers and a representative of the Netherlands Comprehensive Cancer Organization (IKNL) were present. Specialists from the other six DPCG centers were informed on discussed topics by email and agreed. Specific details on best practices were not shared, but extensive background and logistic information was provided, and an elaborate discussion on what best practices should entail was conducted. Ultimately, consensus was reached on the trial design and crucial parts of the three intervention best practices were identified. The shared opinion of the experts was that PACAP-1 should aim for the following points:

- Optimization of patient information and use of chemotherapy
- 70% of patients with a resected tumor should receive adjuvant chemotherapy
- 60% of patients with LAPC should receive chemotherapy
- 40% of patients with metastasized disease should receive palliative chemotherapy
- All pancreatic cancer patients should be discussed in a DPCG or regional multidisciplinary team (MDT), with the exception of a small predefined subgroup (i.e., metastasized patients with WHO performance status III–IV)
- Optimization of PERT and referral to dietician
- Optimization of use of metal stents for biliary drainage

### Randomization, blinding, and treatment allocation {16a} {16b} {16c} {17}

The same randomization order is used as in the PORSCH trial (NCT03400280), a stepped-wedge cluster RCT on the standard of care for postoperative complications after pancreatic surgery, and the PACAP-1 trial, which runs near simultaneously in all DPCG centers in the Netherlands. The reason to use the same randomization order was to obtain an equally long period of optimized standard of care for postoperative complications after pancreatic surgery before switching to the PACAP-1 intervention phase, resulting in homogenous treatment impact throughout centers. Randomization of the 17 pancreatic centers was performed using R statistics software. Stratification was used for center volume of pancreatic resections a year (> 45 vs. ≤ 45). The median value of 45 was based on data from the DPCA 2014–2015). The randomization sequence was unknown to all participating centers and clinicians. Because of the design of PACAP-1, it is not feasible to blind healthcare providers to the best practice treatments and registrations. All PACAP-1 research data are obtained from existing encoded PACAP registries (NCR, DPCA, and PROMs), warranting (pseudo-)anonymization of patients.

### Study procedures {11c}

No specific study procedures are used and no concomitant care and interventions are prohibited during the trial. All best practices are part of current clinical care. PACAP-1 aims to assess the impact of enhanced implementation of current best practices. Therefore, the aim is to improve standard of care compliance by informing, stimulating, and reminding local clinicians per cluster to follow best practice interventions outlined by PACAP-1. Best practice procedures, identified from literature and PACAP, include all interventions documented in the “Intervention phase: best practices” section and Appendix 3 in Table 3. Treatment as usual according to best practice will continue after the study finishes.

### Withdrawal centers {11b}

Because of the stepped-wedge cluster RCT design of PACAP-1, it is important that all randomized DPCG centers complete the trial, so an unequal distribution of patients between current and best practice arms is prevented. However, if a center drops out of the study the randomization order will be maintained. Patients treated in a center that dropped out during this trial will still be accounted for in the final analysis, according to intention-to-treat analysis. If a center stops performing pancreatic surgery, the study will proceed with this center and its referral network.

### Replacement centers after withdrawal

All 17 DPCG centers participate in PACAP-1 and therefore hospitals cannot and will not be replaced after withdrawal.

### Study duration

Planning of the PACAP-1 trial started in PACAP year three (November 2016) and the actual accrual of patients started in May 2018 after obtaining local approval in all participating centers. The implementation phase of the trial will run for 25 months, and the expected implementation end date is July 2020. Follow-up for the primary endpoint will last up until July 2021 and for secondary endpoints up until July 2025.

### Statistical analyses {20a} {20b} {20c}

Outcomes of all patients with pancreatic cancer in the Netherlands will be evaluated before and after the wash-in period (i.e., current practice vs. best practice). Patients will be assigned to current or best practice based on the date of first treatment related to pancreatic cancer (i.e., biliary stent placement, chemotherapy, or primary resection). In case of no treatment or best-supportive care, date of diagnosis will determine assignment to current or best practice. Follow-up time is based on date of diagnosis for all patients. For patients diagnosed in a non-DPCG center, the assignment to current or best practice will depend on the affiliated DPCG center, which will be determined before the start of the study. Primary analysis will be performed with an intention-to-treat analysis according to the randomization order and cross-over dates. If implementation is not performed as scheduled, secondary analysis will be performed according to a per protocol analysis. In the primary analysis, we will use the intention-to-treat principle and patients will be assigned control or intervention according to what was applicable at the time they received their first cancer treatment (i.e., biliary drainage, chemotherapy, or resection). In a secondary per protocol analysis, patients that started in the control period but received part of their cancer treatment during the intervention period will be assigned to the intervention group (e.g., patients who underwent resection in the current practice phase, yet started adjuvant chemotherapy in the best practice phase). Patients diagnosed during the wash-in period will be described but will be excluded from the primary analysis, yet will be included in a secondary analysis. The primary comparison between current and best practice will be performed for patients from all hospitals in the Netherlands. Effect estimates with 95% confidence intervals (CI) will be reported. All *p* values will be based on a two-sided test. *P* values of less than 0.05 will be considered statistically significant.

#### Handling of missing data {20c}

Missing data on baseline characteristics will be imputed by multiple imputation techniques. Outcome data will not be imputed and patients who are lost to follow-up within 1 year will be censored at the date of loss to follow-up. Complete and multiple imputed data analysis will be performed to check for inconsistencies.

#### Baseline characteristics

Descriptive statistics will be used for analysis and reporting of baseline characteristics. Chi-square or Fisher’s exact test will be used to compare categorical variables between patients in current practice and those in best practice. Parametric continuous variables will be reported as mean with standard deviation (SD) and will be compared using the Student’s *t*-test. Non-parametric continuous variables will be reported as median with interquartile range (IQR) and will be compared using the Mann–Whitney U test.

#### Primary outcome {20a}

One-year overall survival will be analyzed with mixed-effects Cox proportional hazards regression models using a random intercept for hospital and a random slope on intervention effect for hospital. The analysis will be adjusted for (calendar) time and for the following baseline characteristics: age at diagnosis and tumor stage at diagnosis using the Union for International Cancer Control (UICC) tumor/node/metastasis (TNM) eighth edition (2018) classification and staging system for pancreatic cancer.

#### Secondary outcomes {20a}

Quality of life will be analyzed using mixed-effects linear regression models, with a random effect per DPCG center. Primary analysis will be performed with area under the curve (AUC) at baseline and follow-up at 3, 6, 9, and 12 months or until death or dropout. Exploratory analysis will be performed with AUC for time points until 3- and 5-year follow-up or until death or dropout, delta analysis, quality adjusted life years (QALY), and for one time point. Adjustment for random and fixed effects will be performed similarly to the primary analysis. Model assumptions will be checked and, if violated, appropriate measures will be taken to derive unbiased standard errors.

Three- and five-year overall survival will be analyzed similarly to the primary endpoint with mixed-effects Cox proportional hazards regression models.

Complication rates will be determined using competing events analysis for time to first complication, corrected for the competing event death. Analyses will be performed for any of all complications and for each type of complication separately. Both cause-specific hazard ratios (reflecting the effect per day alive) and sub-distribution hazard ratios (reflecting the overall effect) will be determined.

Other secondary outcomes will be descriptive in nature, e.g., the proportion of patients in the intervention vs. the control arm using PERT or receiving metal stents.

#### Subgroup and sensitivity analyses {20b}

Subgroup analyses will be performed for three patient subgroups (i.e., patients with resectable, locally advanced, and metastatic pancreatic cancer), two hospital volumes (> 40 vs. ≤ 40 PDs per year [3]) and trial participation in prospective DPCG trials (e.g., PREOPANC-2).

Also, subgroup analysis will be performed for outcomes in pancreatic centers versus referring centers. Patients are allocated to the center in which the primary treatment (e.g., pancreatectomy or first line chemotherapy) has been given.

Sensitivity analyses will be performed for the periods before and after publication of the updated national guideline on pancreatic cancer and European Society of Gastrointestinal Endoscopy guideline on stenting.

#### Interim analysis {21b}

No interim analysis will be performed for study outcomes. A study progression analysis will be performed to assess the number of inclusions at the time point when 50% of inclusions are expected. In the case that < 47.5% of inclusions are acquired at that time point, the length of the steps as described in the “Study design” section will be increased for the remaining time of PACAP-1. As a result, sample size will be reached and statistical power will be maintained. Furthermore, if necessary, when PORSCH increases the length of the steps, PACAP-1 will do so too, to maintain a minimum time difference of 5 months between wash-in phases of both studies in the same cluster.

### Safety reporting {22}

PACAP-1 does not introduce new or experimental interventions. Therefore, this trial is not expected to introduce any additional safety or health risk for patients compared to regular care and hence no specific safety reporting is performed. There is no anticipated harm and compensation for trial participation.

### Handling and storage of data and documents {18a} {18b} {19} {27} {21b}

Data will be collected through DPCA, NCR, and PROMs.

Nationwide DPCA registration, containing mostly surgical data, is completed by local clinicians through an online survey supported by Medical Research Data Management (MRDM). MRDM secures privacy and safe data management and complies with the requirements of information safety with NEN 7510:2011 and ISO 27001:2013 certifications. An opt-out procedure is in place by which patients can refuse the use of their data. Coded DPCA data are securely sent to the PACAP project leader every 3 months. MRDM is the only one with access to the coding key.

NCR data, containing mostly survival, oncological, chemo-, and/or radiotherapy information, are collected from local medical records by trained IKNL registration employees. An opt-out procedure is in place by which patients can refuse the use of their data. Coded NCR data will be obtained from IKNL by the PACAP-1 research team on request. NCR is the only one with access to the coding key.

PROM questionnaires are completed by patients either on paper or online with the first quality of life evaluation at baseline before index treatment. After that, questionnaires will be sent out every 3 months in the first year, every 6 months in the second year, and every 12 months for subsequent years. After collection of paper questionnaires at the AMC, storage and digitization happen at Profiles (subdivision of IKNL focusing on quality of life; https://www.profilesregistry.nl/). Online completed questionnaires are primarily collected at Profiles. Patients sign an informed consent form for participation. The informed consents are available from the corresponding author on request. Coded data will be obtained from Profiles by the PACAP-1 research team at request. Profiles and the PACAP-coordinating investigators are the only ones with access to the coding key.

### Composition of the data monitoring committee and its role and reporting structure {21a}

Because PACAP-1 does not introduce new or experimental interventions and implements best practices from current literature and guidelines on a health care worker level, no data monitoring committee was needed.

### Public disclosure and publication policy {31c} {23} {25}

#### Final manuscript and co-authorship {31a}

PACAP-1 was registered at ClinicalTrials.gov (NCT03513705). The results of PACAP-1 will be submitted to a peer-reviewed journal regardless of study outcome. Co-authorship will be based on the international ICMJE guidelines. Beside the key authors (coordinating investigators as first authors and principal investigators as senior authors), each participating DPCG center will be offered three authorships. Each center will determine who these authors are, but it is advised to include a surgeon, medical oncologist, and gastroenterologist. Additional involved researchers per center can be listed as collaborator.

#### Publications and other studies performed during the trial {5d} {25}

Best practices are based on the current standard of care and literature and identified improvement points from the first years of PACAP. Publications on treatment of pancreatic cancer during the PACAP-1-trial will be reviewed by the PACAP-1 research team. All “practice changing” evidence publications that conflict with the proposed best practices of this trial will be reviewed by the DPCG stakeholders. The DPCG stakeholders and PACAP-1 research team will decide together whether best practices should be adjusted based on the new evidence.

It is expected that several external factors will contribute to the outcomes of PACAP-1. Firstly, the updated Dutch national guideline on diagnosis and treatment of pancreatic cancer and an updated European Society of Gastrointestinal Endoscopy guideline on biliary stenting are expected during our study period. Secondly, national DPCG studies will be developed and executed. For example, the PREOPANC-2 trial on outcomes of neoadjuvant FOLFIRINOX chemotherapy vs. neoadjuvant chemoradiotherapy in patients with resectable and borderline resectable pancreatic cancer has already started including patients. This could influence outcomes of PACAP-1 and will be taken into account in the statistical analyses if possible.

## Discussion

PACAP-1 is a nationwide multicenter randomized controlled stepped-wedge superiority trial with the aim to improve overall survival and quality of life of patients at all stages of pancreatic adenocarcinoma in the Netherlands by enhanced implementation of best-practices.

### Rationale for stepped-wedge cluster randomized design

A structured audit combined with provider feedback, education, outreach visits, and reminders has been shown to be the most effective implementation strategy for change in patients’ care [24]. RCTs are considered the most robust research design for establishing a causal relationship. However, educational interventions at the level of the physician preclude the use of individual randomization due to contamination of the control group. Therefore, a variant of this research method is increasingly used: the stepped-wedge cluster RCT [25]. Data collection in such large multicenter (stepped-wedge) RCTs is, however, often challenging. Therefore, collection through multicenter registries such as PACAP has recently gained interest from researchers as it is a practical way to improve feasibility and at the same time reduce costs for large multicenter RCTs [26].

In a systematic review evaluating 25 studies, it was found that the stepped-wedge cluster RCT design has mainly been applied in evaluating interventions in routine practice [25]. Individual randomization was mostly not deemed possible for the risk of contamination of the control group. Also, using “classic” parallel-group design was not desirable because the PACAP-1 trial aims to implement already previously identified and universally acknowledged “best practices” in the entire population. In a stepped-wedge cluster RCT, clusters (e.g., centers) are randomly allocated a time when they start with the intervention. The order in which the clusters start the intervention is based on a randomization process, thus effectively resulting in a staged implementation in all clusters participating in the trial. This design is especially useful where phased implementation is preferable (e.g., because simultaneous implementation in more clusters is not possible due to logistic reasons), and implementation in all clusters is essential, such as with enhanced implementation of best practices. Additionally, this design makes differentiation from time effects possible, and after calculating the statistical efficiency for PACAP-1, the power achieved with a stepped-wedge cluster RCT was considerably larger than that of a parallel cluster randomized trial.

### Challenges

In the design of the trial, we faced several challenges. First, to avoid contamination in the design of this stepped-wedge trial, only a select group of DPCG experts from every specialty was involved. Although an important aspect of this trial is nationwide support and buy-in, it was actually not desirable to involve a large group of clinicians throughout the country before the actual wash-in phase of their particular center and network. A downside of this could be that there is less involvement and awareness of the trial.

Second, the Netherlands was divided into 17 regions according to the 17 DPCG centers with their respective referral networks. The referral centers usually have one main DPCG center they refer to; however, there might be some cross-over between regions due to geographical reasons, wishes of patients, or other reasons. This will lead to some unavoidable contamination of the trial information.

Third, with the aim to improve survival and quality of life, implementation of a package of best practices, based on nationwide PACAP data, seemed the best strategy. This will, however, make it difficult to determine the effectiveness of each intervention separately. In addition, we advise to include patients in ongoing DPCG trials (e.g., PREOPANC-2) with the similar aim of survival improvement, while the individual trials advise to actively participate in PACAP-1 best practices if already implemented. A measured effect of increased survival may therefore be partly due to the PACAP-1 enhanced implementation and partly due to the different individual trials. PREOPANC-2 is an individually randomized trial and will therefore not suffer from imbalances in patient management due to the PACAP-1 trial. However, if over time the proportion of patients enrolled in PREOPANC-2 changes, this might confound the prognosis of patients in the PACAP-1 trial. To account for this, a sensitivity analysis will be performed, but separate effects can never be measured in detail.

Fourth, every step in this trial, including the wash-in period, accounts for 6 weeks. Therefore, a delay between date of diagnosis or date of resection, and date of commencement of chemotherapy of longer than 6 weeks will lead to an attenuated measurement of the implementation effect. For example, patients who undergo resection a week before the wash-in phase and adjuvant chemotherapy is started 8 weeks after surgery are included in the current-practice group according to intention to treat, yet are treated as the best-practice group. In the Netherlands, median time to adjuvant chemotherapy is 6 to 7 weeks [13], yet due to logistical reasons it was not feasible to prolong steps. To assess the impact of a certain delay, intention to treat as well as per protocol analyses will be performed.

Fifth, the PACAP-1 trial was designed parallel to the PORSCH trial, both concurrent nationwide stepped-wedge trials. PACAP-1 used the identical randomization order as in the PORSCH trial. We considered performing an independent randomization for PACAP-1. However, that would very likely have resulted in unacceptable outcomes: i) possibly both trials would have to implement the same DPCG center simultaneously which is too much information at once and clinicians may lose their trial dedication; ii) multiple combinations of the implementation order per DPCG center would be developed (e.g., first PACAP-1 and second PORSCH or vice versa, or PACAP-1 and PORSCH at the same time), causing bias in trial results, and iii) it ignores the fact that the PORSCH algorithm (or something similar) will probably be the standard of care for postoperative complication management in the Netherlands. Therefore, we believe that PACAP-1 best practices should ideally be implemented in regions that are already in the best practice phase of the PORSCH trial. The possibility to delay the onset of PACAP-1 was deemed unacceptable for a guideline implementation program.

Sixth, during the trial there will be updates of two guidelines in care of pancreatic cancer (i.e., the national guideline on pancreatic cancer diagnostics and treatment and the international ESGE guideline on biliary drainage). This led to more awareness of pancreatic cancer care in current practice and best practice phase centers. As best practice centers are already more attentive, probably the effect of this indirect contamination is larger in current practice centers and may therefore eliminate part of the implementation effect. Sensitivity analyses before and after the publication of both updated guidelines will be performed, but due to attention to these processes over a longer time period, it will be difficult to account for this effect accurately.

Seventh, due to ongoing centralization, centers may stop performing pancreatic surgery. Such centers will, however, remain as oncological centers for patients with non-resectable pancreatic cancer. In such a scenario, the randomization order will not be changed as only 20% of patients undergo a resection and this is according to the intention-to-treat principle.

Eighth, current practice may change during any trial that runs for a longer period of time. In PACAP-1, for example, the advice on adjuvant strategy in the national guideline could change during the trial to modified FOLFIRINOX based on the recent trial by Conroy et al [27]. As modified FOLFIRINOX has been shown to improve survival compared to older chemotherapy regimens, however, this change will likely only positively influence survival in our cohort and therefore may result in biased outcomes.

### Implications and future aims

PACAP-1 is expected to increase awareness and knowledge on best practices and pancreatic cancer care overall, from university pancreatic centers to smaller non-pancreatic centers. This may lead to enhanced implementation of both PACAP-1 best practices and other regional aspects that came to light due to this trial (e.g., the necessity of establishing a regional pancreatic MDT meeting). For this study, a pancreatic cancer team was identified in every region which could lead to improved multidisciplinary communication throughout and between the different networks. This study also identified dieticians in each network. A next step in implementing best practices could be education of all (para-)medical caregivers (e.g., general practitioners, physiotherapist, home care, etc.) to improve awareness and knowledge on pancreatic cancer care.

## Trial status

PACAP-1 was registered with ClinicalTrials.gov on May 1st, 2018 with the identifier NCT03513705. The actual study and recruitment start date was May 22nd, 2018. The estimated recruitment completion date is July 9th, 2020. To date, 13/17 regional networks have undergone the implementation phase and the trial is on schedule.

### Supplementary information

**Additional file 1.** SPIRIT 2013 Checklist for the PACAP-1 trial.

**Additional file 2.** Supplementary materials including details on best practice treatments and -registrations, and additional best practices, and on secondary study endpoints.

## Data Availability

The data that support the findings of this study are available through the scientific committee of the DPCG but restrictions apply to the availability of these data, which were used under license for the current study, and so are not publicly available. Data are, however, available from the authors upon reasonable request and with the permission of the DPCG.

## Appendix 1

### Overview of PACAP projects

*The Dutch Pancreatic Cancer Audit* (*DPCA*)—A clinical audit focusing on surgical patients in all 17 pancreatic cancer centers in the Netherlands. Clinical variables (more than 100 per patient) of all pancreatic resections performed in one of the 17 pancreatic centers in the Netherlands are prospectively registered in the DPCA. In 2014–2015 > 1600 and in 2016 almost 1000 pancreatic resections were registered nationwide. Cross-checks have demonstrated > 90% and > 99% case ascertainment and > 99% and > 99% data accuracy after 1 year and in registry year 2016, respectively.

*The Netherlands Cancer Registry* (*NCR*) hosted by the Netherlands Comprehensive Cancer Organization (IKNL)—A national registry from 1989 onward focusing on all Dutch patients with cancer in which they are registered from diagnosis until death. Including pancreatic cancer in all Dutch hospitals (DPCG and referring centers), detailed clinical data of patients receiving chemotherapy, radiotherapy, or no treatment are obtained by trained IKNL registration employees in every Dutch hospital.

*The Dutch Pancreas Biobank* (*PancreasParel*)—PancreasParel obtains blood and tissue samples from all patients with pancreatic and periampullary cancers. The biobank is part of the Parelsnoer Institute (www.parelsnoer.org). Preoperative blood samples, perioperative tissue samples (tumor tissue and normal tissue), and postoperative blood samples are collected. Since its official launch in February 2015, over 488 patients have been included. Currently, 13 centers participate in the biobank, including four academic centers and one teaching hospital. IRB approval has been obtained in six more centers and logistic facilities are currently being established in these hospitals. For PACAP-1, no additional biological specimens will be collected.

*Patient Reported Outcome Measures* (*PROMs*)—PROMs are prospectively registered for all patients with pancreatic and periampullary cancer starting in the winter of 2015. After 7 months, seven academic and 11 peripheral centers in the Netherlands had joined this initiative. Within 18 months, 517 patients were included and 308 patients returned quality of life questionnaires (i.e., response rate 60%).

*An online expert panel*—Online expert panel meant to provide advice on resectability of pancreatic cancer. The PACAP expert panel received 180 patients from nine centers, referred between April 2015 and July 2017. Sub-analysis of the first 79 referrals identified locally advanced pancreatic cancer (LAPC) in 100% of cases and in 51% (40/79) of patients there was an additional treatment or a change in the planned treatment strategy. Of these patients, a resection with curative intention was performed in eight patients (10%) and 28 patients (35%) were included in a clinical trial, investigating local ablative therapies. In all cases the expert panel advice was provided within 1 week.

## Appendix 2

### Methods of implementation of PACAP-1 best practices

To achieve effective implementation of PACAP-1 best practices, a structured wash-in phase is designed:

1. At the start of the wash-in phase, a regional “kick-off” evening is organized by the PACAP-1 research team at the DPCG center with presentations on details of the interventions and logistics of PACAP-1. All involved physicians and nurses from the DPCG-center and peripheral hospitals in that region are invited.
2. At this evening, the regional pancreatic cancer team is introduced as a central group to implement the best practices, PACAP-1 interventions, and logistics in that region.
3. Also, all PACAP-1 support materials will be made available. They include the detailed protocol, the PACAP-1 smartphone application, decision support tools, pocketsize PACAP-1 overview, and access to protected parts of www.pacap.nl.
4. In the first and second weeks of the wash-in phase, introductory presentations will be given to each medical specialty. The PACAP-1 research team will also participate in a local MDT meeting in which pancreatic cancer patients are discussed.
5. In weeks 3–6 of the wash-in phase, the PACAP-1 research team will discuss the progress of the implementation with the regional pancreatic cancer team and involved clinicians and nurses from peripheral hospitals. With this approach, identified points of improvement in the implementation strategy will be adjusted if necessary.

Once a DPCG center and that region is in the best practice phase, reminder visits will be scheduled and reminder emails will be sent:

1. A 2-monthly update will be sent via email to the involved clinicians and nurses with a graph that shows the “scores” for compliance with PACAP-1. Other DPCG-centers will be anonymized in the graph.
2. Four to six months after the wash-in phase, a reminder visit will be scheduled with presentations on the progress of PACAP-1. This provides local clinicians and nurses the opportunity to ask questions.
3. If necessary, more update and reminder visits will be scheduled.

Throughout PACAP-1, the regional pancreatic cancer teams or the PACAP-1 research team will be available for questions from anyone involved in this study.

## Appendix 3

.

**Table 3:** List of PACAP-1 interventions per medical specialty

**Medical oncology**				
	**Intervention**	**Definition**	**Outcome**	**Measurement**
**1**	Standard information and decision support tool	Use of standard information and decision support tool for all pancreatic cancer patient subgroups (e.g., via https://bit.do/beslisboom)	Survival	NCR
**2**	Discussion on chemotherapy (resectable patients)	Percentage of resectable pancreatic cancer patients with whom Chemotherapy options are discussed in DPCG center	Survival Quality of life	NCR PROMs DPCA
**3**	Diagnostics LAPC patient established in DPCG center	Percentage of LAPC patients in the diagnostic phase that are discussed in DPCG MDT meeting	Survival Quality of life	NCR PROMs DPCA
**4**	Post-induction chemotherapy discussion of LAPC patient in DPCG center	Percentage of LAPC patients treated with chemotherapy that are discussed in DPCG MDT meeting after 2 months of therapy	Survival Quality of life	NCR PROMs DPCA
**5**	PERT	Percentage of patients with EPI who receive PERT	Survival Quality of life	NCR PROMs
**6**	Key parameter WHO performance status reporting	Percentage of patients with a (suspected) pancreatic malignancy, in whom the WHO performance status is reported at first presentation.	–	NCR DPCA
**7**	Pre-treatment pathology confirmation	Percentage of patients with (suspected) locally advanced and metastatic pancreatic cancer, with histological or cytological proof of pancreatic adenocarcinoma	–	NCR
**8**	PROMs	Percentage of patients with a (suspected) pancreatic malignancy who are registered for the PACAP PROMs	–	PROMs
**9**	Biobanking	Percentage of patients receiving pancreatic resection for suspected malignancy who are registered for the PancreasParel	–	PancreasParel
**Surgery**				
	**Intervention**	**Definition**	**Outcome**	**Measurement**
**1**	Medical oncology referral	Percentage of patients with pancreatic cancer referred to medical oncologist for consultation on adjuvant chemotherapy	Survival Quality of life	NCR PROMs
**2**	PERT	Percentage of patients with EPI who receive PERT	Survival Quality of life	NCR PROMs
**3**	Synoptic discharge letter	Percentage of patients receiving pancreatic resection for a (suspected) malignancy in whom the synoptic complication table is used in the discharge letter	–	DPCA
**4**	Synoptic POC	Percentage of patients undergoing pancreatic resection in whom the synoptic POC is used in the operation report	–	DPCA
**5**	PROMs	Percentage of patients receiving pancreatic resection for (suspected) malignancy who are registered for the PACAP PROMs	–	PROMs
**6**	Biobanking	Percentage of patients receiving pancreatic resection for (suspected) malignancy who are registered for the PancreasParel	–	PancreasParel
**7**	Standardized complication management	Standardized approach to early detection and treatment of pancreatic fistula (PORSCH trial)	Postoperative complications	DPCA PROMs PORSCH
**Gastroenterology**				
	**Intervention**	**Intervention**	**Outcome**	**Measurement**
**1**	Metal stent	Percentage of patients with a (suspected) pancreatic malignancy requiring biliary drainage receiving a metal (rather than a plastic) stent	Complications	NCR DPCA
**2**	PERT	Percentage of patients with EPI who receive PERT	Survival Quality of life	NCR PROMs
**3**	Pre-treatment pathology confirmation	Percentage of patients with (suspected) locally advanced and metastatic pancreatic cancer with histological or cytological proof of pancreatic adenocarcinoma	–	NCR
**Pathology**				
	**Intervention**	**Definition**	**Outcome**	**Measurement**
**1**	Synoptic reporting	Percentage of patients receiving pancreatic resection for a suspected malignancy in whom the resection specimen is recorded according to the PALGA/Dutch Society of Pathology nationwide synoptic report	Number of R1 resections	DPCA
**Radiology**				
	**Intervention**	**Definition**	**Outcome**	**Measurement**
**1**	Synoptic reporting	Percentage of patients with a (suspected) pancreatic in whom the computed tomography (CT) is recorded according to the Dutch Society of Radiology CT-checklist	–	DPCA

Abbreviations: *NCR* Netherlands Cancer Registry, *PROMS* patient reported outcome measures, *DPCA* Dutch Pancreatic Cancer Audit, *DPCG* Dutch Pancreatic Cancer Group, *LAPC* locally advanced pancreatic cancer, *MDT* multidisciplinary team, *PERT* pancreatic enzyme replacement therapy, *EPI* exocrine pancreatic insufficiency, *WHO* World Health Organization, *PACAP* Dutch Pancreatic Cancer Project, *POC* postoperative conclusion, *PALGA* nationwide network and registry of histo- and cytopathology of the Netherlands

## Abbreviations

ASA: American Society of Anesthesiologists
CAC: Cluster autocorrelation
DICA: Dutch Institute for Clinical Auditing
DPCA: Dutch Pancreatic Cancer Audit
DPCG: Dutch Pancreatic Cancer Group
EPI: Exocrine pancreatic insufficiency
ICC: Intracluster correlation coefficient
IKNL: Netherlands Comprehensive Cancer Organization
LAPC: Locally advanced pancreatic cancer
MDT: Multidisciplinary team
NCR: Netherlands Cancer Registry
PACAP: Dutch PAncreatic CAncer Project
PALGA: Nationwide network and registry of histo- and cytopathology of the Netherlands
PancreasParel: Dutch Pancreatic Biobank
PD: Pancreatoduodenectomy
PERT: Pancreatic enzyme replacement therapy
POC: Postoperative conclusion
PORSCH: POstopeRative Standardization of Care: THe Implementation of Best Practice After Pancreatic Resection
PROMs: Patient-reported outcome measures
UICC: Union for International Cancer Control
WHO: World Health Organization
