# Supplementary material for: Impact of nationwide enhanced implementation of best practices in pancreatic cancer care (PACAP-1): a multicenter stepped-wedge cluster randomized controlled trial
Source: Trials. 2020 Apr 16;21:334. doi: 10.1186/s13063-020-4180-z (PMC7161112; doi:10.1186/s13063-020-4180-z)
Supplement: Supplementary file 2 — Additional file 2. Supplementary materials including details on best practice treatments and -registrations, and additional best practices, and on secondary study endpoints. [file 13063_2020_4180_MOESM2_ESM.doc]

**SUPPLEMENTARY MATERIALS**

**Treatment-1: Optimal patient information on chemotherapy**

The identified points of improvement were discussed with an advisory committee containing 7 medical oncologists from DPCG centers.

*Best-practice-treatments – concerns medical oncologists, surgeons and gastroenterologists*

Decision support tool – An information and decision support tool for three pancreatic cancer subgroups (see below) are designed to be used for patient and clinicians treatment decisions (<https://bit.do/beslisboom>). Practical patient information lines are provided.

Resectable cancer – All resectable patients will be referred to the medical oncologist in the DPCG center in which they are operated in for information on adjuvant treatment options. Per DPCG center, a medical oncologist with focus on pancreatic cancer will see the referred patients. Treatment can be given either in the DPCG center or in a peripheral center.

Details on chemotherapy choice and guidance in treatment decisions are provided with the information and decision support tool (<https://bit.do/beslisboom>).

LAPC - Primary assessment of all LAPC patients will take place in the DPCG center MDT meeting to establish a treatment plan. Treatment can be given either in the DPCG center or in a peripheral center. Every LAPC patient treated in a DPCG center or peripheral center with chemotherapy will be reevaluated after 2 months of treatment in the MDT meeting of the DPCG center to assess possible treatment change and resectability.

Details on chemotherapy choice and guidance in treatment decisions are provided with the information and decision support tool (<https://bit.do/beslisboom>).

Metastasized disease – All metastasized patients will be discussed in the MDT meeting of a DPCG center or in a regional MDT where at least one physician of a DPCG center is present, with the exception of a predefined subgroup (by expert consensus: metastasized patients with WHO performance status 3-4, see chapter ‘National expert meeting’).

Details on chemotherapy choice and guidance in treatment decisions are provided with the information and decision support tool (<https://bit.do/beslisboom>).

**Background Treatment-1: Optimal patient information on chemotherapy**

It is widely reported that adjuvant and palliative chemotherapy for resectable, locally advanced and metastasized pancreatic cancer patients provides significant survival benefit and improvement in quality of life^1-7^. According to the Dutch national guidelines on pancreatic cancer all patients with good WHO performance status after pancreatic resection should be advised to receive adjuvant chemotherapy and in case of locally advanced or metastasized disease palliative chemotherapy^8^. However, national DPCA data from these 3 years showed that 36% WHO 0-1 pancreatic cancer patients did not receive adjuvant chemotherapy. NCR data from 2005-2013 showed that approximately 10-15% of pancreatic cancer patients were eligible for resection (M0-resected patients), 30-40% were M0-not resected patients and 50-55% were metastasized (M1) patients (Figure 1). Median percentage of M0 not-resected patients receiving chemotherapy was 14%, with an increase from 10% in 2005-2007 to 18% in 2011-2013 (unpublished data NCR). This group consisted of locally advanced pancreatic cancer (LAPC) patients, but also of patients that were not resected because of high age (> 80 years) and bad WHO performance status (≥ WHO 2). Still, the majority of patients were not treated according to the guideline.

The median percentage of patients with metastasized pancreatic cancer receiving palliative chemotherapy was 23%, with an increase from 13% in 2005 to 30% in 2013. Of these patients, 8.4% died within 30 days of start of first line chemotherapy^9^. In 2015 in the Netherlands, 10% of patients with stage 4 pancreatic cancer started with chemotherapy in the last month of life and 11% received last chemotherapy dose in the last 14 days before death (unpublished data NCR). Of all patients with metastasized pancreatic cancer, diagnosed between 2005-2013, 26% died within 30 days after diagnosis^9^. This can partly explain why 70% of M1 patients did not receive palliative chemotherapy, but for the majority of these patients palliative chemotherapy should be considered. In addition, a study performed in the Netherlands showed that hospital volume of palliative chemotherapy for metastatic pancreatic cancer was associated with improved survival^10^. This indicates the presence of regional differences in treatment and outcomes.

A percentage of patients will have made a grounded decision to not be treated with chemotherapy. However, other patients possibly did not receive chemotherapy due to lack of (understanding of) information or after referral back to a regional center after diagnosis. For this latter group it is essential to improve informing of patients in an expert center to increase the amount of patients with good WHO performance status that receive chemotherapy to not only improve time to recurrence and survival, but also quality of life. Moreover, with the aim to optimize use of chemotherapy, the percentage of patients that start new chemotherapy treatment in the last month of life and patients that receive the last dose in the last 4 weeks of life should be reduced to a minimum.


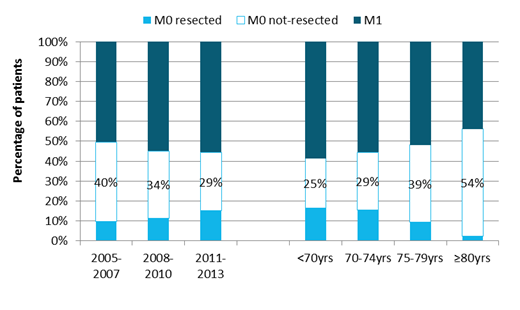


**Figure 1.** M0 and M1 patients from NCR data 2005-2013 (unpublished data)

**Treatment-2: Pancreatic enzyme replacement therapy (PERT) in case of exocrine pancreatic insufficiency (EPI)**

This best-practice has been developed with nutritional experts in the field. Standardized questions have been developed for clinicians to assess the presence of malnutrition and support the optimal use of pancreatic enzymes.

*Best-practice-treatment – concerns medical oncologists, surgeons and gastroenterologists*

At every outpatient clinic visit, patients will be asked about EPI symptoms and PERT will be prescribed if necessary. It is advised to refer patients to a dietician if PERT is prescribed or if unintended weight loss (without EPI) is present. Depending on hospital logistics, some centers will choose to describe PERT to all new pancreatic cancer patients or at least all patients that underwent pancreatic surgery. After a few months of treatment it can be evaluated whether PERT treatment is still necessary. A pocked sized information sheet with EPI symptoms, advise on dietician referral and start dosage of PERT is developed.

Pancreatic enzyme-application for patients

A mobile application focusing on EPI and PERT has been developed, as supportive material for patients. Patients can enter their daily complaints and their diet. In return, the application gives an advice on PERT dosage and whether a patient should contact their dietician or physician. This application will be offered to all patients with EPI and PERT.

**Background Treatment-2: Pancreatic enzyme replacement therapy (PERT) in case of exocrine pancreatic insufficiency (EPI)**

EPI occurs in up to 90% of patients after pancreatic resection and in 25-50% with LAPC^11-13^. In inoperable patients EPI occurs in up to 92% after two months after diagnosis^14^. Steatorrhea and weight loss are the most common manifestations of EPI, with potentially large effects on quality of life and nutritional status^15^. EPI is grossly underdiagnosed and undertreated^11^. PERT is effective in treating EPI^16^. Optimal treatment with PERT requires referral to a dietician for evaluation of individually adjusted dosages per meal or snack and patient education. Recent studies showed that use of PERT was independently associated with improved survival following pancreatoduodenectomy for cancer^17, 18^. Therefore, with attention for EPI and adequate treatment, nutritional status, quality of life and survival can improve.

In general practice the diagnosis of EPI is mainly based on presence of steatorrhea, weight loss or abdominal complaints. However, the reference standard for the diagnosis of EPI is the coefficient of fat absorption (CFA)^19, 20^. This measurement involves a specific diet with 72-hour stool collection, which is a burden for patients, logistically challenging and expensive. Another option is the fecal elastase-1 (FE-1) test, which only requires one stool sample and is less expensive. Literature however is controversial about the FE-1 test as a diagnostic tool^21-24^. It described that the vast majority of patients after pancreatoduodenectomy develop EPI, and therefore PERT should be recommended to all postoperative patients, even without performing diagnostics^25^. Within the PACAP-1 trial, it is advised to start PERT in all patients with pancreatic cancer (or at least those after pancreatic surgery) or in all patients with symptoms of EPI, because 1) literature is inconclusive on the additional value of the FE-1 test, 2) the CFA is too much of a burden, 3) the prevalence of EPI is high for all patient subgroups, and 4) a simple and effective treatment (i.e. PERT) is available.

**Treatment-3: Optimal biliary drainage**

This best practice involves the optimal, evidence-based, strategy for biliary drainage in patients with obstructive jaundice caused by pancreatic cancer.

*Best-practice-treatment – concerns gastroenterologists*

Patients with pathologically confirmed pancreatic cancer requiring biliary drainage will receive a metal stent. PACAP-1 aims for a proportion of ≥75% metal stents in patients with pancreatic cancer.

Indications for biliary drainage with metal stent for extrahepatic biliary obstruction for the different patient subgroups are:

- Resectable tumor
  - Bilirubin >250 µmol/L
  - Cholangitis
  - Symptomatic obstructive jaundice (e.g. pruritis)
  - Before neoadjuvant chemotherapy if bilirubin > 25 µmol/L
  - Bilirubin <250 µmol/L and waiting time for surgery > 3 weeks (preference early surgery)
- Irresectable tumor (LAPC or metastasized disease)
  - Cholangitis
  - Symptomatic obstructive jaundice (e.g. pruritis)
  - Before start of neo-adjuvant or palliative chemotherapy if bilirubin > 25 µmol/L
  - In case elective plastic stent exchange is due it should be replaced with metal stent

Only if placement of a metal stent is not possible due to anatomy (e.g. close relation to the hilum) or if prior severe complications after metal stent placement like cholecystitis occurred, a plastic stent is an acceptable alternative.

In case of extrahepatic biliary obstruction requiring drainage, but without pathologic confirmation, the preferred stent is a fully covered metal stent.

**Background Treatment-3: Optimal biliary drainage**

Preoperative biliary drainage with metal stents is related to a lower number of stent related complications (i.e. cholangitis) and less stent dysfunction (e.g. re-obstruction and migration) as compared to plastic stents^26^. Cholangitis, stent dysfunction and the resulting inadequate biliary drainage will lead to worsened patient condition and delayed treatment with chemotherapy or surgery. However, the use of plastic stents in patients requiring preoperative biliary drainage is still frequent. During the first three PACAP years, 35% of stents in the Amsterdam UMC (location AMC) was plastic, including plastic stents that were placed previously in a referral center. NCR data (unpublished) from 2015 of all patients with pancreatic cancer, show that firstly placed stents were plastic in 39% of the cases, metal in 40% and unknown in 21%. Type of stent is added as variable in the DPCA since 2017 and in this year 54% of 165 stents placed in pancreatic cancer patients that underwent resection in the Netherlands was plastic. In 2016 and 2017, almost 50% of patients with a solid tumor on radiographic studies and registered in the DPCA, underwent preoperative biliary drainage^27^.

A recent RCT studied effectiveness and costs for plastic and, uncovered and partially covered self-expandable, metal stents for palliation of extrahepatic bile duct obstruction. This study showed that both types of metal stents had longer functional time than plastic stents. Although metal stents initially were more expensive, total costs after 1 year did not differ between the different types of stent^28^. In addition, a recent study investigated cost-effectiveness of metal vs. plastic stents in patients with LAPC or metastatic pancreatic cancer with a life expectancy of more than 6 months. This study demonstrated that placement of metal stents at initial onset of obstructive jaundice reduced the need for stent revision and was a more cost-effective strategy than plastic stents, while improving quality of life^29^.

Furthermore, the updated European Society of Gastrointestinal Endoscopy guideline on biliary stenting recommends the use of self-expandable metal stents for biliary obstruction of known etiology; preoperatively and for palliation of extrahepatic malignant biliary obstruction^30^.

Compliance and stent related complications will be measured using the DPCA in patients requiring preoperative biliary drainage. In (non-)resectable patients this will be measured through the NCR.

**Best-practice-registration-1: Use of the checklist for radiology reports – concerns radiologists**

The radiology checklist will be used for the report of all CT-scans during the diagnostic process of pancreatic cancer patients in all DPCG centers.

**Background Registration-1: Use of the checklist for radiology reports**

The radiology checklist for reporting pancreatic cancer imaging has been developed by the Dutch association for radiology and the DPCG. Although advised by the DPCG, DPCA data from January-June 2017 show that the CT checklist was only used in 61/143 (43%) of the cases.

**Best-practice-registration-2: Use of standardized table with intra-operative events in operation report and complications of surgical treatment in discharge letters – concerns surgeons**

*Standardized postoperative conclusion*

A synoptic postoperative conclusion table has also been developed by the DPCG. This will be used in every operation report of a pancreatic resection in all DPCG centers.

*Standardized discharge report*

A synoptic discharge report following pancreatic surgery has been developed by the DPCG. This report will be used in every discharge letter of patients that underwent pancreatic resection in all DPCG centers.

**Background Registration-2: Use of standardized table with intra-operative events in operation report and complications of surgical treatment in discharge letters**

The standardized postoperative conclusion (POC) table for the operation report and table of complications of surgical treatment for the discharge letters are developed by the DPCG. Both tables have been tested previously and facilitate better registration of treatment and outcome. However, DPCA data from January-June 2017 showed that standardized tables are not often used, although advised by the DPCG:

1. Standardized POC table: in 89/143 (62%) cases used
2. Standardized complication table: 40/143 (28%) cases used

**Best-practice-registration-3: Use of nationwide PALGA standard for reporting pancreatic cancer pathology - concerns pathologists**

The synoptic report by the DPCG and PALGA is advised as standardized postoperative pancreatic pathology report.

**Background Registration-3: Use of nationwide PALGA standard for reporting pancreatic cancer pathology**

The use of synoptic pathology reports has been associated with an improved histopathological assessment^31, 32^. A synoptic report of pancreatic pathology has been developed by the DPCG and the nationwide pathology network and registry (PALGA). PACAP-1 will measure the percentage of patients receiving pancreatic resection for a suspected malignancy, in who the resection specimen is recorded according to the synoptic report and correlate this to the number of R1 resections both recorded in the DPCA. However, DPCA data from January-June 2017 showed that PALGA report is only used in 46/143 (32%) of the cases.

**Best-practice-registration-4: Report of WHO performance status – concerns medical oncologists, surgeons and gastroenterologists**

WHO performance status will be reported at first presentation of patients with (suspected) pancreatic cancer.

**Background Registration-4: Report of WHO performance status**

Performance status (WHO) is an important characteristic of patients with a (suspected) pancreatic cancer. For example, the new FOLFIRINOX chemotherapy has demonstrated significant improvement in survival in patients with metastatic pancreatic cancer, however due to an increase in toxicity compared to standard gemcitabine it is reserved for patients with a maximum WHO performance status of 1. From January-June 2017, the WHO performance status was reported in the DPCA in 126/143 (88%) of the cases. In the NCR, the WHO performance status was reported in 57% of 2149 patients diagnosed in 2017 with pancreatic cancer who did not underwent resection of the primary tumour (versus 49% of 1880 in 2015).

**Additional best practices**

*PACAP PROMs registry – concerns medical oncologists, surgeons and gastroenterologists*

Each patient with a pancreatic malignancy is eligible for the PACAP PROMs and will be asked to participate before start of primary treatment (preferably) or before start of new treatment episode.

All patients with a pancreatic or periampullary malignancy are eligible (all tumor stages) for the PACAP PROMs. Questionnaire time points are at baseline and follow up at 3, 6, 9, 12, 18, 24, 36 months and yearly thereafter. In 18 months over 500 patients were registered for inclusion in the PACAP quality of life questionnaire study. Overall, response rates are approximately 60%. With almost 2400 newly diagnosed patients per year in the Netherlands, a significant amount of patients are not registered to participate in PACAP PROMs.

*Biobanking (PancreasParel)*

Each patient with a pancreatic tumor is eligible for participation in the PACAP PancreasParel as described in APPENDIX 1. Currently not all Dutch pancreatic centers participate in the PancreasParel. Therefore, implementation of PancreasParel in more centers is encouraged. As biobanking is a component of PACAP and is stimulated within PACAP-1, it is reported briefly. However, because blood and tissue samples are collected to be subjected to novel research techniques in the future, results will be reported separately from PACAP-1.

*Pathologic analysis (PA) in patients with (suspected) metastatic and locally advanced pancreatic cancer - concerns medical oncologists and gastroenterologists*

Pathologic confirmation of all patients with (suspected) metastatic and locally advanced cancer will be performed.

According to the Dutch pancreatic cancer guideline, all patients with (suspected) metastatic pancreatic cancer should receive cytologic or histopathologic confirmation. This is especially important prior to palliative chemotherapy, as cytologic or histologic proof of another tumor type may impose large differences in treatment, survival and quality of life. In 10% of M1 patients^9^ and 17% of M0-not resected patients (NCR data unpublished) cytologic or histopathologic confirmation is not obtained prior to palliative chemotherapy for (suspected) metastatic pancreatic cancer.

*Postoperative complication management*

Approximately 20% of patients with pancreatic cancer are amenable to resection. Pancreatic resection is associated with high risk of postoperative complications of 50%^33^. A common complication is pancreatic fistula that can lead to life-threatening situations if not managed adequately^34^. Therefore, the ‘POstopeRative Standardization of Care: THe Implementation of Best Practice After Pancreatic Resection’ or PORSCH-trial is designed (NTR6905). The objective of this nationwide trial in the Netherlands is *to investigate if the implementation of a best practice algorithm for postoperative care focusing on early detection and step-up management of postoperative pancreatic fistula results in a lower rate of major complications and death after pancreatic resection as compared to current practice.* As the PORSCH-trial also includes all 17 DPCG centers and has the same stepped-wedge design as the PACAP-1 trial, both studies will be executed in a parallel manner. Because PORSCH aims to improve postoperative outcomes within 90 days and PACAP-1 aims to improve long-term outcomes, results will be reported separately. For detailed information on postoperative complication management, we refer to the PORSCH-trial protocol.

*Participation in DPCG randomized controlled trials – concerns all healthcare providers in DPCG centers*

PACAP-1 aims to obtain a higher participation rate of eligible patients in DPCG supported randomized trials, such as the next RCT (PREOPANC-2), which started in 2018. In 2017 the PREOPANC-1, a DPCG randomized clinical trial was closed after including all 244 patients. In this study, preoperative radiochemotherapy versus immediate surgery for resectable and borderline resectable pancreatic cancer was investigated^35^. This study was considered the most important oncological study of the DPCG, which could improve the outcome of pancreatic cancer patients. Although the accrual met the requirements, not all DPCG centers included sufficient eligible patients as might be expected. Just three centers were responsible for more than 60% of all included patients.

With the help of the DPCG, the PACAP-1 team will support better trial participation. Furthermore, together with principal investigators, the PACAP-1 team will present an overview of included patients in all DPCG centers during our return visits and will contact centers when inclusion stays behind. By these measures we aim to include more patients in a shorter period of time and a better participation of all centers in this open randomized control clinical trials with primary objective progression free and overall survival.

**Support - PACAP-1 smartphone application**

To support and moderate the enhanced implementation of above described best practices, a PACAP-1 smartphone application will be made available to all healthcare providers at start of the wash-in period of their cluster. This is an informative application that provides a summary of key best practices that are implemented during PACAP-1.

**Secondary study endpoints**

*Intervention outcomes:*

- Quality of life at baseline and all follow-up moments (3, 6, 9, 12, 18, 24, 36 months and yearly thereafter, until death or drop out)
  - EQ-5D-5L
  - EORTC QLQ-C30
  - EORTC QLQ-PAN26
  - EPI questionnaire
- 3- and 5-year overall survival
- Complications will be measured during the complete duration of the PACAP-1 trial:
  - Chemotherapy (palliative or (neo)adjuvant)
    - Toxicity grade 3-4
    - Type of toxicity (hematological, gastrointestinal, neurological, other)
  - Stent placement (metal or plastic)

*Process measure outcomes*

- Proportion of post-pancreatectomy patients receiving adjuvant chemotherapy
- Proportion of patients receiving neoadjuvant chemotherapy
- Proportion of LAPC patients that underwent pancreatic resection
- Proportion of unresectable patients receiving palliative chemotherapy
- Proportion of patients that received palliative chemotherapy in last month of life
- Proportion of patients with suspected or confirmed EPI receiving PERT
- Proportion of patients with suspected or confirmed EPI that visited a dietician
- Proportion of patients requiring biliary drainage receiving a metal stent
- Proportion of (suspected) metastasized patients undergoing pathological analysis

*Registry outcomes:*

- Proportion of diagnosed pancreatic cancer patients registered for PROMs
- Proportion of diagnosed pancreatic cancer patients registered in DPCA
- Proportion of post-pancreatectomy patients with synoptic discharge letter
- Proportion of post-pancreatectomy patients with postoperative conclusion table
- Proportion of patients with (suspected) unresectable pancreatic cancer with documented WHO performance status at first presentation
- Proportion of post-pancreatectomy patient with synoptic resection specimen report
- Proportion of patients diagnosed with a solid pancreatic tumor with CT-scan checklist
- Proportion of patients registered for biobanking in participating PancreasParel centers
- Proportion of LAPC patients discussed in regional Multidisciplinary Team meeting during diagnostic period
- Proportion of treated LAPC patients that underwent resection after chemotherapy
- Proportion of LAPC patients discussed in Multidisciplinary Team meeting 2 months after start of chemotherapy
- Use of smartphone application

*Other study parameters*

- Proportion of patients included in other DPCG prospective trials
- Baseline patient characteristics:
  - Age
  - Sex
  - Height in cm
  - Weight in kg
  - WHO performance status
  - Relevant medical history
    - Disease requiring medical treatment, such as cardiovascular disease, renal failure, pulmonary disease, diabetes
  - American Society of Anesthesiologists (ASA) classification
  - Pre-treatment pathology diagnosis
  - Tumor stage at diagnosis

**REFERENCES**
